# Jasmonate response decay and defense metabolite accumulation contributes to age-regulated dynamics of plant insect resistance

**DOI:** 10.1038/ncomms13925

**Published:** 2017-01-09

**Authors:** Ying-Bo Mao, Yao-Qian Liu, Dian-Yang Chen, Fang-Yan Chen, Xin Fang, Gao-Jie Hong, Ling-Jian Wang, Jia-Wei Wang, Xiao-Ya Chen

**Affiliations:** 1 National Key Laboratory of Plant Molecular Genetics, CAS Center for Excellence in Molecular Plant Sciences, Institute of Plant Physiology and Ecology, Shanghai Institutes for Biological Sciences, University of CAS, Chinese Academy of Sciences, Shanghai 200032, People's Republic of China; 2 School of Life Science and Technology, ShanghaiTech University, Shanghai 200031, People's Republic of China; 3 State Key Laboratory of Breeding Base for Zhejiang Sustainable Pest and Disease Control, Institute of Virology and Biotechnology, Zhejiang Academy of Agricultural Sciences, Hangzhou 310021, People's Republic of China; 4 Plant Science Research Center, Shanghai Key Laboratory of Plant Functional Genomics and Resources, Shanghai Chenshan Botanical Garden, Shanghai 201602, People's Republic of China

## Abstract

Immunity deteriorates with age in animals but comparatively little is known about the temporal regulation of plant resistance to herbivores. The phytohormone jasmonate (JA) is a key regulator of plant insect defense. Here, we show that the JA response decays progressively in *Arabidopsis*. We show that this decay is regulated by the miR156-targeted SQUAMOSA PROMOTER BINDING PROTEIN-LIKE9 (SPL9) group of proteins, which can interact with JA ZIM-domain (JAZ) proteins, including JAZ3. As SPL9 levels gradually increase, JAZ3 accumulates and the JA response is attenuated. We provide evidence that this pathway contributes to insect resistance in young plants. Interestingly however, despite the decay in JA response, older plants are still comparatively more resistant to both the lepidopteran generalist *Helicoverpa armigera* and the specialist *Plutella xylostella*, along with increased accumulation of glucosinolates. We propose a model whereby constitutive accumulation of defense compounds plays a role in compensating for age-related JA-response attenuation during plant maturation.

## Abstract (web summary)

Immunity deteriorates with age in animals but less is known about how aging affects immunity in plants. Here, Mao *et al*. show that responsiveness to the major insect defense hormone JA declines as plants age, but insect resistance still increases as plants accumulate secondary metabolites such as glucosinolates.

As sessile organisms, plants have evolved complex defense systems against herbivores for successful survival and reproduction. Induced defense response refers to immune responses elicited by specific stimuli whereas constitutive defense refers to the accumulation of insecticidal components in plant tissues during the course of normal growth and development^1^. Plants of *Arabidopsis thaliana* produce glucosinolates (GLSs), which function as defense metabolites against insect herbivores and pathogens^2^.

In plants, JA is the major defense hormone in activating defense reactions against herbivorous insects and necrotrophic pathogens, and in *Arabidopsis* most responses are regulated by the JA-amino acid conjugate jasmonoyl-L-isoleucine (JA-Ile)^3^,^4^. JA ZIM-domain (JAZ) proteins, which are the repressors of JA signalling, have two conserved domains: the N-terminal ZIM domain and the C-terminal Jas domain^5^,^6^. The Jas domain is a protein–protein interaction surface required for binding to either transcription factors such as MYC2, or CORONATINE INSENSITIVE1 (COI1), a component of the ubiquitin E3 ligase SCF^COI1^ and the JA-Ile receptor^7^,^8^,^9^. In normal conditions, the relatively high levels of JAZ proteins repress the activity of transcription factors. External stimuli, such as wounding or insect attack, cause JA-Ile concentration to rapidly rise in plant cells, which triggers COI1-JAZ interaction and degradation of JAZs by the 26S proteasome^10^, releasing transcription factors to activate downstream defense genes^11^. Therefore, the abundance of JAZ proteins in cells, determined largely by the rates of protein degradation, controls the output of JA response^12^. Interestingly, protein stability of MYC transcription factors is also involved in JA response^13^,^14^.

Plant defense involves a metabolic cost, where a tradeoff occurs between defense and growth^15^. In animals immunosenescence is a common phenomenon^16^. In plants, immunity is also associated with age, as old plants may display increased resistance to pathogens, which is referred to as age-related resistance^17^. For plant-herbivore interactions, the Plant Vigour Hypothesis is based on observations that many herbivores attack young and vigorous plants more frequently than old and mature plants^18^. However, the molecular mechanisms underlying ARR and the Plant Vigour Hypothesis remain elusive.

In plants, miR156 functions as an important regulator of age-dependent development through targeting a group of transcription factors called SQUAMOSA PROMOTER BINDING PROTEIN-LIKE (SPL)^19^. The level of miR156 is high during the juvenile stage and steadily decreases during later plant growth and development, leading to a progressive increase in the level of SPLs, which regulate a broad range of processes including flowering^20^,^21^, secondary metabolite production^22^,^23^, trichome initiation^24^,^25^,^26^, vernalization^27^,^28^ and shoot regeneration^29^. The phytohormone gibberellin (GA) plays an important role in regulating diverse aspects of plant growth and development^30^,^31^. Similar to JAZ proteinss in the JA signalling pathway, DELLAs, a group of GRAS family proteins, function as repressors to control GA signal output. When the level of bioactive GAs increases, DELLAs are subject to ubiquitination and degradation^30^. Recent investigations revealed that GA signalling cross-talks with both the JA response and the miR156-SPL mediated aging pathways. DELLAs directly bind to SPL proteins and interfere with their transcriptional activity^32^, and they also interact with JAZ proteins or MYC2 to modulate the JA response^33^,^34^.

Here we demonstrate that the JA response declines with plant age. We show that this attenuated JA response is primarily regulated by the miR156-targeted SPL proteins that can interact with certain JAZ proteins, and appears to be independent of the GA signalling pathway. We propose a model whereby accumulation of GLS in older plants accumulates for attenuated JA response.

## Results

### The JA response declines with plant age

**Figure 1:**
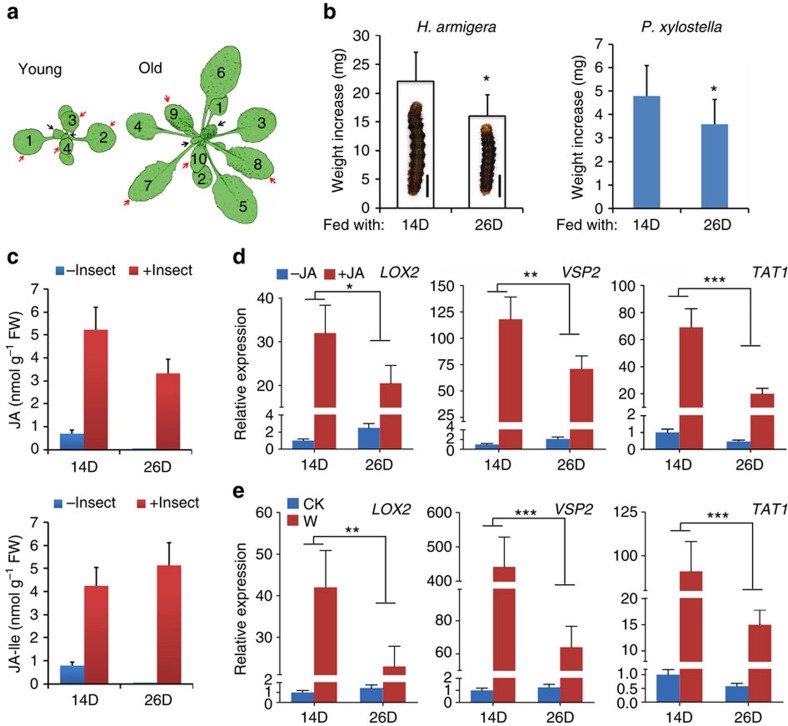
Insect resistance enhances and JA response attenuates with plant age. (**a**) Diagrams of young (14-day-old, 14D) and old/adult (26-day-old, 26D) plants of *Arabidopsis thaliana* (Col-0) grown in LD. Black arrows indicate the newly initiated leaves (new leaves, ∼3 mm in width), and red arrows indicate the rapidly expanding leaves. (**b**) Weight increase of *H. armigera* and *P. xylostella* larvae fed with rapidly expanding leaves harvested from the indicated plants for 3 days, both gained less weight from the old (26D) plants. Data are means ±s.d. (*n*=25), asterisk indicates significant difference from the 14D group (Student's *t*-test, *P<0.05). Embedded in the column is the image of the *H. armigera* larva after feeding. Scale bar, 1 cm. (**c**) Analysis of JA and JA-Ile contents in young (14D) and old (26D) plants by UPLC-MS. Plants were challenged by *H. armigera* third instar larvae for 12 h and the rapidly expanding leaves were collected for analysis. Intact plants were used as control. Data are means ±s.d.(*n*=3). (**d**,**e**) Expression of *LOX2*, *VSP2* and *TAT1* in young (14D) and old (26D) plants in LD. Transcript levels were detected by qRT-PCR. Data were analysed by multiple comparisons (Tukey test) followed by two-way ANOVA (*P<0.05, **P≤0.01, ***P≤0.001). Error bars represent ±s.d. (*n*=3). (**d**) Gene expressions in total aerial tissues of young (14D) and old (26D) plants 4 h post-MeJA treatment, the JA response attenuated with plant age. The expression in the 14D control plants (−JA) was set to 1. (**e**) Gene expressions in rapidly expanding leaves of the young (14D) and the old (26D) plants 2 h post-wounding (W) treatment, the wounding response attenuated with plant age. The expression in the 14D intact plants (CK) was set to 1. ANOVA, analysis of variance; UPLC-MS, ultra performance liquid chromatography-mass spectrometry.

Cotton bollworm (*Helicoverpa armigera*) is a generalist lepidopteran pest that can live on a wide range of plants including *Arabidopsis*^35^,^36^. When *H. armigera* larvae were placed on 14- and 26-day-old^2^ *Arabidopsis* plants for three days, respectively, the larvae on young plants grew faster than those on old plants. Another lepidopteran, Diamondback moth (*Plutella xylostella*), is a specialist herbivore living on plants of Brassicaceae, and *Arabidopsis thaliana* can be used as a model host^37^. When *P. xylostella* larvae were tested, similar results were obtained: young plants provided quicker larval growth than old plants (Supplementary Fig. 1). At vegetative stage, *Arabidopsis* produces new leaves continuously. To focus on plant age, and minimize the impact of leaf developmental state, we used detached leaves at the rapidly expanding stage for feeding assays (Fig. 1a). Again, the larvae of *H. armigera* and *P. xylostella* gained more weight from the young plant leaves than old plant leaves (Fig. 1b). Thus, results from intact plants and detached leaves both suggest that plants become more resistant to insect herbivores as they age.

The JA signalling pathway plays important roles, not only in plant defense against herbivorous insects, but also in a wide range of developmental processes^38^,^39^,^40^. To test if JA response was altered according to plant age, we first analysed the endogenous levels of JA and the bioactive JA-Ile. Although higher in young than in old plants, the JA and JA-Ile contents were generally low in untreated plants. Upon *H. armigera* damage, their levels were evidently elevated, but the resultant JA-Ile contents did not show a significant difference between the young and the old plants (Fig. 1c). We then monitored the expression of JA-inducible genes, including *LIPOXYGENASE 2* (*LOX2*/*AT3G45140*), *VEGETATIVE STORAGE PROTEIN 2* (*VSP2*/*AT5G24770*) and *TYROSINE AMINOTRANSFERASE* (*TAT1*/*AT4G23600*)^4^,^41^. After application of methyl-JA (MeJA), expression of JA-responsive genes in aerial tissues was induced to a higher degree in young plants compared with old plants (Fig. 1d). When the newly initiated leaves (new leaves) were analysed, the results were similar; young plants exhibited a more pronounced JA response than old plants (Supplementary Fig. 2a). We next performed wounding treatments to mimic insect damage and to trigger the JA response. Although expression of all three genes was drastically elevated in leaves of both young and old plants after wounding, the latter exhibited a much weaker (half to several folds lower) induction (Fig. 1e). Together, these data demonstrate that, although JA-Ile levels do not exhibit a clear tendency of age-dependent change, both wounding and JA responses deteriorate during plant growth, opposite to the gradual strengthening of insect resistance.

### SPL9 negatively regulates JA response and insect resistance

**Figure 2:**
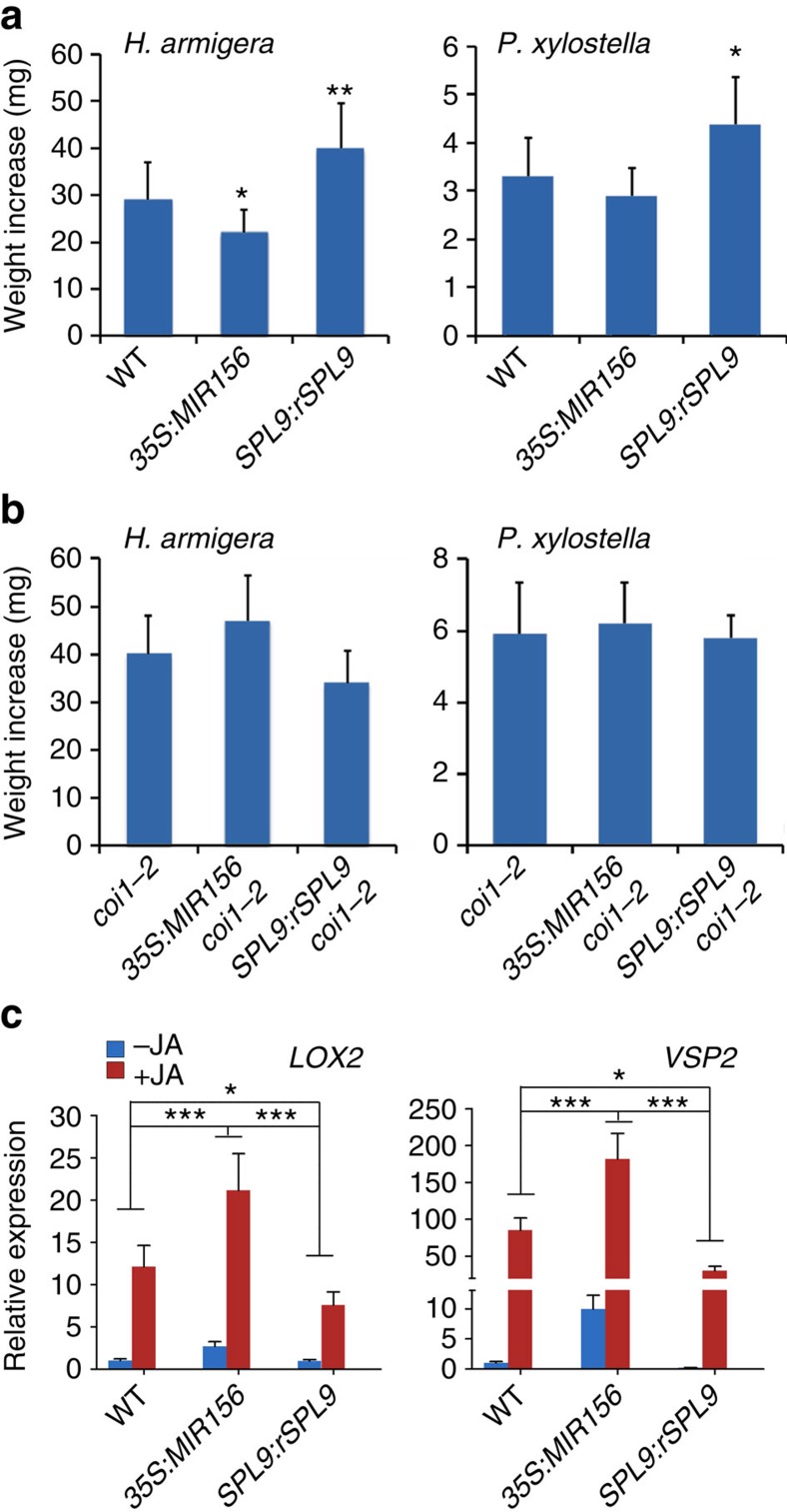
SPL9 negatively regulates plant resistance to insect and JA response. (**a**,**b**) Weight increase of *H. armigera* and *P. xylostella* larvae fed with the indicated plant leaves, both insects gained higher weight from the *SPL9:rSPL9* plants. Leaves from the ∼30-day-old plants in SD were used in feeding. Data are means ±s.d. (*n*=25), asterisks indicate a significant difference from the wild-type (WT) group (Student's *t*-test, *P<0.05, **P<0.01). (**c**) *LOX2* and *VSP2* expressions in plants over-expressing miR156 (*35S:MIR156*) or SPL9 (*SPL9:rSPL9*). Plants (30-day-old in SD) were treated with 50 μM MeJA (+JA) or ethanol (−JA) as control, and 4 h later the transcript levels in new leaves of the indicated plants were detected by qRT-PCR. The expression in the wild-type was set to 1. Data were analysed by multiple comparisons (Tukey test) followed by two-way ANOVA (*P<0.05, ***P≤0.001). Error bars represent ±s.d. (*n*=3). ANOVA, analysis of variance.

The miR156-SPL module regulates plant maturation. Overexpression of miR156 (*35S:MIR156*) prolongs the juvenile stage, whereas inhibition of miR156 activity by target mimicry (*35S:MIM156*) or expression of a miR156-resistant form of *SPL9* (*SPL9:rSPL9*) accelerates the display of adult traits^20^,^21^. We compared insect resistance of wild-type, *35S:MIR156*, *SPL9:rSPL9* and *35S:MIM156* plants. As both *SPL9:rSPL9* and *35S:MIM156* plants flower extremely early in LD, producing less than three rosette leaves, we grew the plants in short days (SD) to prolong the vegetative stage. As in LD, the wild-type plants in SD also exhibited a reduced JA response at a later growth stage (Supplementary Fig. 2b). Interestingly, we observed a negative effect of the miR156-regulated SPLs on plant resistance. For *H. armigera*, the *SPL9:rSPL9* and *35S:MIM156* leaves provided larvae a higher weight increase, whereas the *35S:MIR156* leaves retarded larval growth. For *P. xylostella*, the larvae also grew faster on *SPL9:rSPL9* leaves than on wild-type or *35S:MIR156* leaves (Fig. 2a and Supplementary Fig. 3a). These data suggest that, although functioning as an aging cue, the miR156-targeted SPLs repress plant defense against insects, contrary to the observation that the old plants had higher insect resistance than young plants (Fig. 1b).

To test whether SPL9 affected plant resistance to insects by interfering with JA signalling, *35S:MIR156* and *SPL9:rSPL9* were introduced into the JA-insensitive mutant *coi1-2*, respectively. The *SPL9* transcript level was elevated in *SPL9:rSPL9 coi1-2* and reduced in *35S:MIR156 coi1-2* (Supplementary Fig. 4), as expected. Insect feeding assays showed that both *H. armigera* and *P. xylostella* larvae grew faster on *coi1-2* than on the wild-type leaves (Fig. 2a,b), as reported for *Pieris rapae*, another lepidopteran herbivore^42^. However, the negative effect of SPL9 on insect resistance was abolished in the *coi1-2* background (Fig. 2b), suggesting that JA signalling was involved in the repression of defense by *SPL9*, although a possible indirect influence due to different developmental or physiological states because of the changed level of SPLs could not be excluded. Consistent with their insect resistance, the *35S:MIR156* plants exhibited a higher JA response than the wild-type plants, whereas the *SPL9:rSPL9* and *35S:MIM156* plants were less sensitive to the MeJA treatment (Fig. 2c, Supplementary Figs 3b,c and 5).

**Figure 3:**
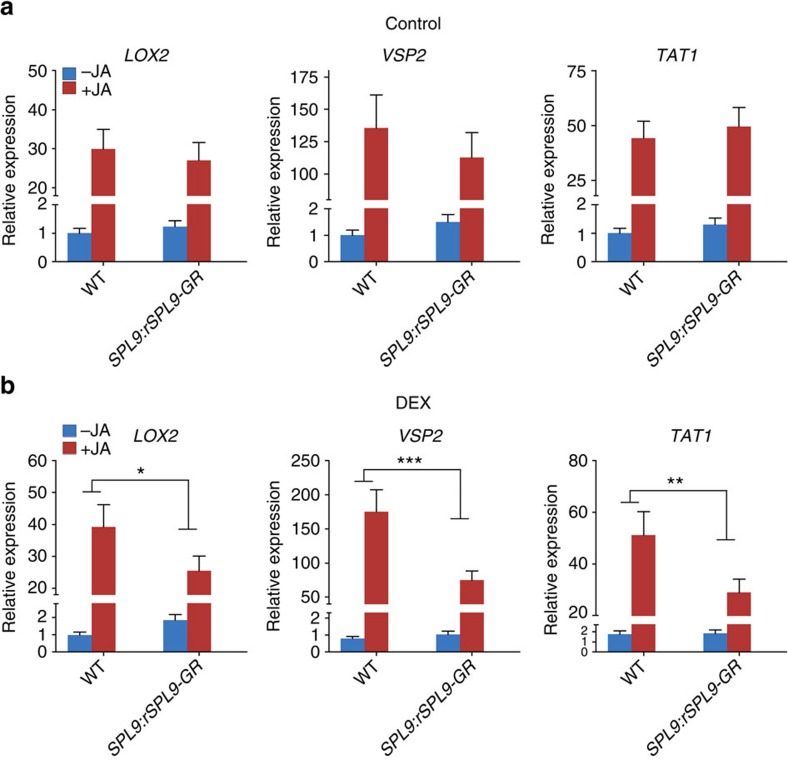
Translocation of SPL9 into nucleus by DEX treatment dampens JA response. The wild-type and *SPL9:rSPL9-GR* plants (12D in LD) were first sprayed with ethanol (control) (**a**) or 10 μM DEX (**b**), and after 12 h the plants were treated with 50 μM MeJA (+JA) or ethanol (−JA). Four hours later the transcript levels in the first pair of leaves were detected by qRT-PCR. The expression in the wild-type free from DEX and JA was set to 1. Data were analysed by multiple comparisons (Tukey test) followed by two-way ANOVA (*P<0.05, **P≤0.01, ***P≤0.001). Error bars represent ±s.d. (*n*=3). ANOVA, analysis of variance.

To analyse the effect of SPL9 on JA response more directly, we employed an inducible expression system. In *SPL9:rSPL9-GR* plants, rSPL9 was fused to the hormone-binding domain of the rat glucocorticoid receptor^43^ and expressed under the *SPL9* promoter. Treating the plant with dexamethasone (DEX) leads to translocation of rSPL9-GR fusion protein from cytoplasm to nucleus and a phenotype similar to that of the *rSPL9* expressing (*SPL9:rSPL9*) plant^20^. We found that, in the absence of DEX, the response to MeJA was similar between the *SPL9:rSPL9-GR* and the wild-type plants (Fig. 3a). Upon DEX-application, the *SPL9:rSPL9-GR* plants became less sensitive to MeJA, as the degree of induction of all three JA-responsive genes tested was reduced (Fig. 3b). When the DEX treated plants were used for feeding assays, both *H. armigera* and *P. xylostella* larvae on the *SPL9:rSPL9-GR* leaves gained slightly but significantly more weight than those on the wild-type leaves. However, without DEX treatment the larval growth was similar between the *SPL9:rSPL9-GR* and the wild-type groups (Supplementary Fig. 6). These data suggest a direct involvement of SPL9 in dampening JA response and insect resistance. Furthermore, because the *SPL9:rSPL9-GR* plants were grown in LD (Fig. 3) whereas the *SPL9:rSPL9* and *35S:MIM156* plants were grown in SD (Fig. 2c, Supplementary Figs 3 and 5), these results also imply that the negative effect of the miR156-targeted SPLs on JA response occurred in both the LD or SD growth conditions that we employed.

### SPL9 can interact with JAZ proteins

**Figure 4:**
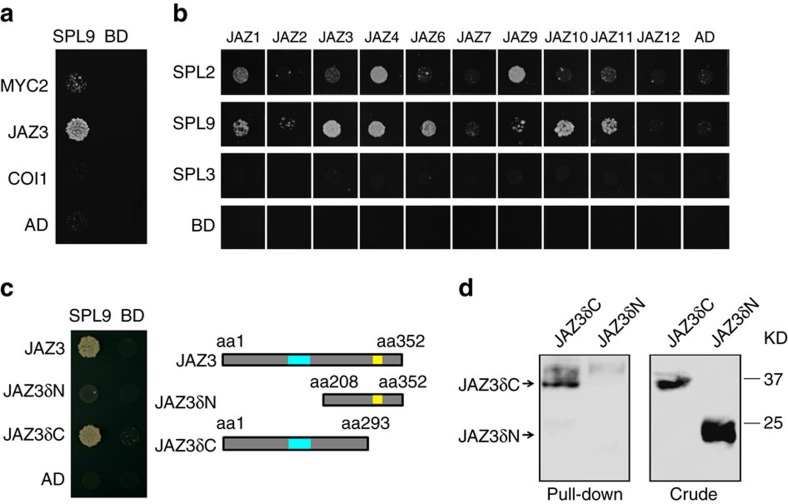
SPL proteins can interact with JAZ proteins. Yeast two-hybrid assay. SPLs were fused to GAL4 DNA-binding domain (BD), MYC2, COI1, JAZ3, JAZ3δN and JAZ3δC was fused to GAL4 activation domain (AD), respectively. Interactions were examined with 10 mM (for SPL2 and SPL3) or 15 mM (for SPL9) 3-amino-1,2,4-triazole. Schematic diagrams of truncated versions of JAZ3 are shown in **c**, blue box indicates ZIM domain and yellow box indicates Jas domain. SPL9 and SPL2, but not SPL3, interacted with JAZs (**a**,**b**), and the N-terminal of JAZ3 was responsible for binding to SPL9 (**c**). (**d**) Pull-down assay of JAZ3-SPL9 binding. Recombinant HIS-SPL9 protein was incubated with total proteins of the tobacco leaf expressing either JAZ3δN-HA or JAZ3δC-HA driven by the 35 S promoter. Anti-HA antibody was used to detect the truncated fusion proteins of JAZ3 before (Crude) or after (Pull-down) immunoprecipitation. KD, kilodalton.

SPL9 has dual molecular functions, acting as transcriptional activator^20^,^21^ or as a signalling modulator through binding other factors^22^,^29^. Quantitative real-time PCR (qRT-PCR) showed that the elevated *SPL9* level did not upregulate *JAZ* genes at the transcriptional level (Supplementary Fig. 7). We then asked whether SPL9 attenuates the JA response through interacting with known JA signalling factors, such as COI1, MYC2 and JAZ, at the protein level. In yeast two-hybrid assays SPL9 had no obvious interactions with COI1 and MYC2 (Fig. 4a), but a direct interaction was observed between SPL9 and a number of JAZ proteins, including JAZ1, JAZ3, JAZ4, JAZ6, JAZ10 and JAZ11. SPL2, a homologue of SPL9, interacted with JAZ1, JAZ4 and JAZ9. In contrast, SPL3, which belongs to a separate SPL group and harbours the SBP DNA-binding domain only^44^, did not bind to any of the JAZ proteins tested (Fig. 4b). Yeast two-hybrid and pull-down assays further demonstrated that JAZ3δC (JAZ3 without the Jas motif-containing C-terminal) interacted with SPL9, whereas removal of the ZIM domain-containing N-terminal of JAZ3 (JAZ3δN) abolished the binding activity (Fig. 4c,d and Supplementary Fig. 8).

### SPL9 promotes JAZ3 accumulation

**Figure 5:**
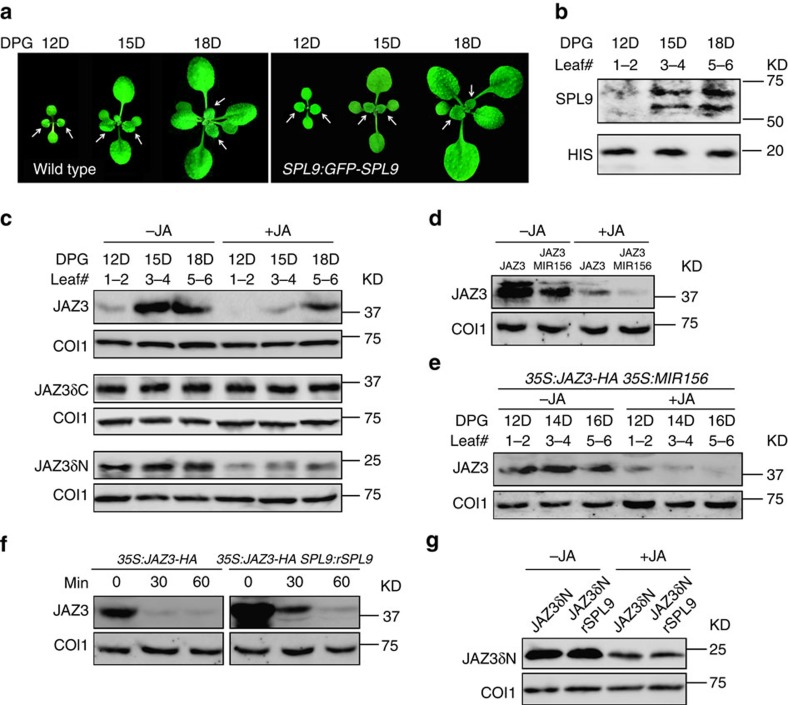
SPL9 promotes JAZ3 protein accumulation. (**a**) Images of wild-type and *SPL9:GFP-SPL9* plants in LD at indicated days post germination (DPG). White arrows indicate new leaves (leaf #1–2, 3–4 and 5–6). (**b**–**g**) Protein levels of SPL9 and JAZ3 in new leaves. KD, kilodalton. (**b**) SPL9-GFP fusion protein in new leaves as described in **a** were detected by anti-GFP antibody, the protein level increased with plant age. (**c**–**g**) JAZ3-HA accumulation with plant age. Plants in LD were treated with 50 μM MeJA (+JA) or ethanol (-JA), and new leaves were collected one hour later or at the indicated time post treatment for immunoblotting. JAZ3-HA or the truncated versions were detected using anti-HA antibody. COI1 in each sample was detected using anti-COI1 antibody. (**c**) JAZ3-HA (top), JAZ3δC-HA (middle) and JAZ3δN-HA (bottom) proteins in leaves (leaf #1–2, 3–4 and 5–6) harvested from *35S:JAZ3-HA*, *35:JAZ3δC-HA* and *35S:JAZ3δN-HA* plants at the indicated DPG. The JAZ3-HA fusion protein, but not its truncated versions, exhibited the age-dependent accumulation. (**d**) JAZ3-HA level was decreased in the *35S:MIR156* background. The first pair of leaves from *35S:JAZ3-HA* (JAZ3) and *35S:JAZ3-HA 35S:MIR156* (JAZ3 MIR156) plants were collected for analysis. (**e**) JAZ3-HA level was similar among leaves (leaf #1–2, 3–4 and 5–6) harvested from *35S:JAZ3-HA 35S:MIR156* plants at the indicated DPG. (**f**) JAZ3-HA level was increased in the *SPL9:rSPL9* background. The first pair of leaves from *35S:JAZ3-HA* and *35S:JAZ3-HA SPL9:rSPL9* plants were analysed. (**g**) JAZ3δN-HA protein level in *35S:JAZ3δN-HA* and *35S:JAZ3δN-HA SPL9:rSPL9* plants, which was uncoupled from SPL9.

As plants grow, *SPL9* transcript abundance is gradually elevated as miR156 levels decline^20^. To examine the change of SPL9 protein abundance during plant age, we used *SPL9:GFP-SPL9* plants where a green fluorescent protein (GFP)-SPL9 fusion was expressed from its endogenous promoter. New leaves (#1–2, 3–4 and 5–6) of *SPL9:GFP-SPL9* were collected sequentially during plant growth (Fig. 5a), and the levels of GFP-SPL9 fusion protein were detected using an antibody against GFP. The GFP-SPL9 was undetectable in total protein extracts. We then isolated the nucleoproteins for blotting, which showed a positive signal band of the expected size, in addition to a smaller fragment likely caused by protein degradation. Indeed, the GFP-SPL9 protein level increased gradually along with plant age (Fig. 5b).

Analysis of *JAZ3* gene expression showed that its transcript level in the first three pairs of leaves did not change significantly (Supplementary Fig. 9a). However, the JAZ3 protein exhibited an age-dependent accumulation: in *JAZ3:JAZ3-FLAG* plant the JAZ3 fusion protein was undetectable in the first two pairs of leaves, but became evident thereafter (Supplementary Fig. 9b). Similar increase of the protein abundance was observed when the *35S* promoter was used to drive *JAZ3-HA* expression (Fig. 5c), further suggesting that this age-related temporal pattern was not conferred by gene transcription. In response to JA, COI1 recruits JAZ proteins for degradation^10^. However, we did not detect changes in the COI1 protein level with age (Fig. 5 and Supplementary Fig. 9b), suggesting that JAZ3 accumulation in old plants was not due to varying levels of COI1. In *35S:JAZ3δC-HA* plants, JAZ3δC-HA was resistant to MeJA treatment (Fig. 5c), consistent with the fact that the Jas motif in the C-terminal is indispensible for COI1-directed degradation^5^,^6^,^9^. Intriguingly, the level of JAZ3δC-HA was age-insensitive, being constant among the sequentially initiated leaves (Fig. 5c), suggesting that plant age may regulate JAZ3 stability via COI1-directed degradation. In *35S:JAZ3δN-HA* plants, the N-terminal truncated JAZ3 was also insensitive to plant age, although still responded to MeJA (Fig. 5c), raising the possibility that the N-terminal of JAZ3 contains the sensor that recognizes the plant age cue.

The fact that JAZ3 can bind to SPL9 prompted us to test whether SPL9 has a role in this process. We generated *35S:JAZ3-HA 35S:MIR156*, *35S:JAZ3-HA SPL9:rSPL9* and *35S:JAZ3δN-HA SPL9:rSPL9* plants by crossing. Neither *35S:MIR156* nor *SPL9:rSPL9* affected *JAZ3* transcript levels in the *35S:JAZ3-HA* or *35S:JAZ3δN-HA* plants (Supplementary Fig. 10). However, in *35S:JAZ3-HA 35S:MIR156* plants, in which the expression of *SPL* genes was downregulated, JAZ3-HA protein abundance was drastically reduced and no longer elevated with plant age (Fig. 5d,e and Supplementary Fig. 11 ), suggesting the involvement of miR156 in controlling JAZ3 protein accumulation. In contrast, in *35S:JAZ3 SPL9:rSPL9* plant the high level of SPL9 promoted JAZ3-HA accumulation to a much higher level, which remained detectable even after MeJA treatment (Fig. 5d and Supplementary Fig. 12). Clearly, the increased level of SPL9 was accompanied by elevated JAZ3 accumulation.

Importantly, in *35S:JAZ3δN-HA SPL9:rSPL9* plants, the JAZ3δN-HA protein became insensitive to the high level of SPL9 (Fig. 5g), further supporting the role of the N-terminal of JAZ3 in mediating the SPL9-induced JAZ3 accumulation and consistent with the notion that the SPL9-JAZ3 interaction may be relevant as JAZ3δN cannot interact with SPL9. Yeast three-hybrid assays showed that the COI1-JAZ3 interaction was weakened by the increased amount of SPL9 (Supplementary Fig. 13). Together, these results suggest that SPL9 stabilizes JAZ3 by preventing COI1-mediated protein degradation, possibly via binding to its N-terminal end.

### GA does not disrupt age-related JA response attenuation

**Figure 6:**
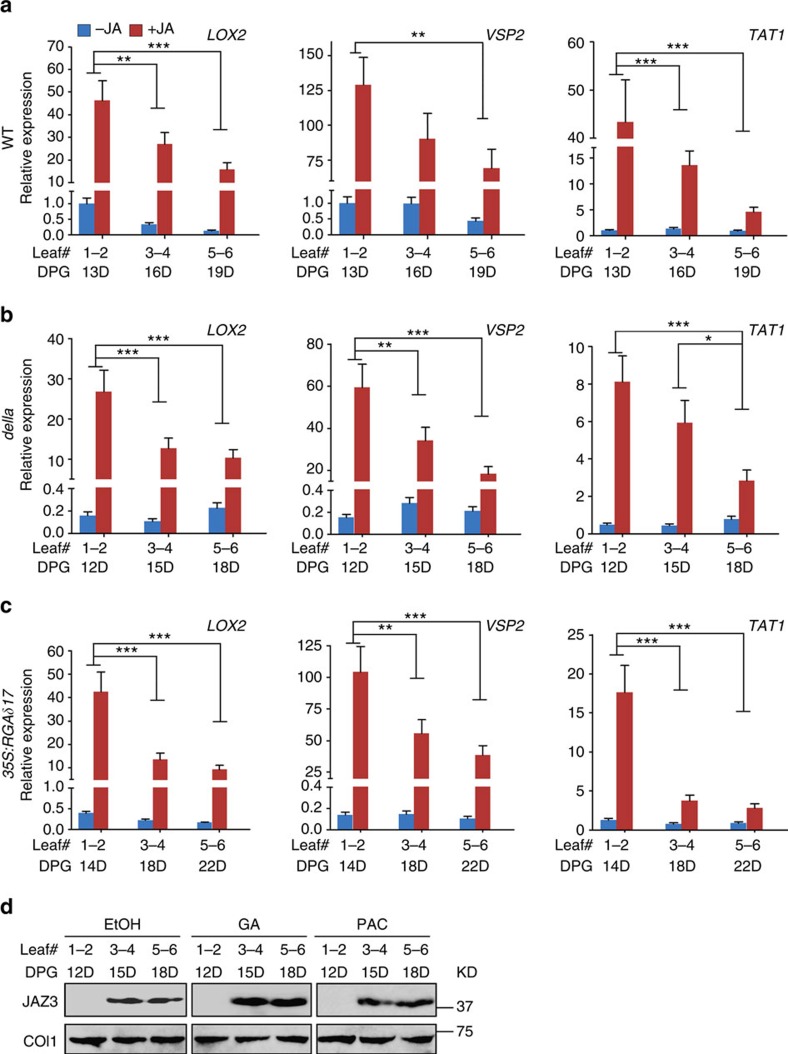
The age-dependent JA response attenuation is not altered by GA. qPCR analyses of the transcript levels of *LOX2*, *VSP2* and *TAT1* in leaves of the wild-type (WT) (**a**), the penta *della*-deficient mutant (*della*) (**b**) and the DELLA over-expressor (*35S:RGAδ17*) (**c**) plants. All plants are in Ler-0 background. Plants in LD condition were treated with 50 μM MeJA (+JA) or ethanol (−JA) as control, new leaves (leaf #1–2, 3–4 and 5–6) were harvested at the indicated DPG and analysed. The expression in the first pair of leaves (leaf #1–2) of the wild-type control (−JA) plants was set to 1. Data were analysed by multiple comparisons (Tukey test) followed by two-way ANOVA (*P<0.05, **P≤0.01, ***P≤0.001). Error bars represent ±s.d. (*n*=3). (**d**) JAZ3 protein accumulation in GA and PAC treated plants. Ten days after germination, the LD grown *35S:JAZ3-HA* plants were sprayed with 50 μM GA or PAC one time per day, and the new leaves were harvested 2 h after the treatment at indicated days (D) post germination. JAZ3-HA was detected using anti-HA antibody. COI1 in each sample was detected using anti-COI1 antibody. ANOVA, analysis of variance; KD, kilodalton.

The plant growth hormone GA has been shown to interfere with JA signalling^33^,^45^,^46^. DELLAs are negative regulators of the GA signalling pathway. The *A. thaliana* genome encodes five DELLA proteins: RGA, GAI, RGL1, RGL2 and RGL3. In a pentuple mutant of all five DELLA genes (*della*), downstream genes of the GA pathway are constitutively upregulated; by contrast, *35S:RGAδ17* plants, in which the mutated *RGA* over-accumulates due to the lack of the motif required for GA-induced protein degradation, are much less sensitive to GA^47^. When the pentuple mutant *della* and the DELLA over-expressor *35S:RGAδ17* were treated with MeJA, both maintained similar age-dependent declines in JA response as seen in the wild-type plant (Fig. 6a–c and Supplementary Fig. 14), although an overall weakened induction of the JA-responsive genes was observed in the *della* mutant, which agrees with the report that GA suppresses JA signalling output^33^. These data suggest that GA signalling, while affecting the magnitude of JA response, does not impair the general tendency of plant age-controlled JA signal deterioration.

To further examine the influence of GA, the *35S:JAZ3-HA* plants were daily-treated with GA (to reduce the DELLA protein level) or paclobutrazol (PAC, to block GA biosynthesis). Immunoblot assays revealed that neither GA nor PAC treatments blocked the JAZ3 protein accumulation in old plants (Fig. 6d). Together, these data suggest that altered GA levels do not reverse the progressive attenuation of JA response with plant age.

### High levels of JAZ3 attenuate plant insect resistance

To analyse the role of JAZ3 in insect resistance, we chose two *35S:JAZ3-HA* lines, among which *35S:JAZ3-HA-2* had a much higher level of JAZ3-HA than *35S:JAZ3-HA-1* (Supplementary Fig. 15a). As expected, JA response was inversely correlated to the level of JAZ3: the *35S:JAZ3-HA-2* plant exhibited less sensitivity to MeJA treatment than the wild-type and the *35S:JAZ3-HA-1* plants (Supplementary Fig. 15b,c). Insect feeding assays further showed that *H. armigera* larvae fed *35S:JAZ3-HA-2* leaves gained significantly more weight than those fed wild-type or *35S:JAZ3-HA-1* leaves (Supplementary Fig. 15d), indicating that elevated accumulation of JAZ3 protein reduced plant resistance to insects.

### Glucosinolate accumulation strengthens insect resistance

**Figure 7:**
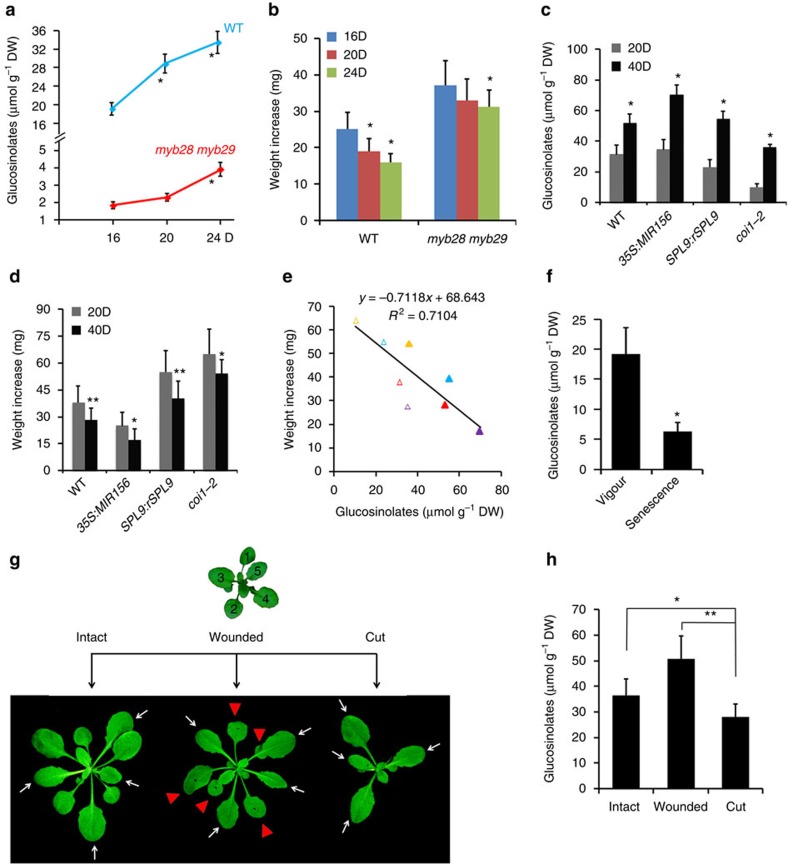
Increased accumulation of GLSs in old plant enhances insect resistance. GLSs content was detected by LC-MS (**a**,**c**,**f**,**h**). Error bars represent ±s.d. (*n*=3). (**a**) GLSs content in the wild-type (Col-0) and *myb28 myb29*. Rapidly expanding leaves of 16-, 20- and 24-day-old plants in LD were used. Asterisk indicates significant difference from the 16-day-old plant (Student's *t*-test, *P<0.05). (**b**) Weight increase of *H. armigera* larvae fed with leaves described in **a**. Error bars represent ±s.d. (*n*=25), asterisk indicates significant difference from the 16D group (Student's *t*-test, *P<0.05). (**c**) GLSs content in rapidly expanding leaves of 20- (20D) or 40-day-old (40D) plants of different genotypes in SD. Asterisk indicates significant difference from the 20D group (Student's *t*-test, *P<0.05). (**d**) Weight increase of *H. armigera* larvae fed with leaves described in **c**. Error bars represent ±s.d. (*n*=25), asterisk indicates significant difference from the 20D group (Student's *t*-test, *P<0.05, **P<0.01). (**e**) Negative relation between GLSs content and larval growth. *X* axis represents GLSs content in the plant leaves as in **c** and *y* axis indicates larval weight increase in **d**. Weight increase of *H. armigera* larvae fed with leaves of 20-day-old plant in SD of the wild type (WT), *35S::MIR156*, *SPL9::rSPL9* and *coi1-2* shown as red, purple, blue and yellow hollow triangles, respectively, and those with 40-day-old plant leaves as the respective solid triangles. (**f**) GLSs decline in leaves during senescence. The first two leaves at vigorous stage (Vigour) from the 14-day-old plants or at senescent (Senescence) stage from the 28-day-old plants in LD were used. Asterisk indicates significant difference from vigour stage (Student's *t*-test, *P<0.05). (**g**,**h**) Possible mobilization of GLSs from the early leaves to new leaves. The first to fifth leaves from the 20-day-old plant in LD were removed or wounded, four days later the rest four leaves from the plant (cut), the same set of leaves from wounded plants (wounded) and the intact plant (intact) (**g**) were harvested for GLSs detection (**h**). White arrows indicate leaves collected for analysis and red triangles refer to wounded leaves. Asterisk indicates significant difference (Student's *t*-test, *P<0.05, **P<0.01).

The above results suggest that the miR156-SPL-JAZ module is responsible for the age-dependent decay of JA responsiveness. However, in feeding assays, old plants were more resistant to insects than young plants (Fig. 1b and Supplementary Fig. 1). There are of course many other factors than JA that affect plant defense. Plant secondary metabolites can act as phytoalexins against herbivores and pathogens, and in *Arabidopsis* GLSs serve as major defensive compounds to deter generalist insects^2^,^48^. We then performed both gas-chromatography-mass spectrometry and liquid chromatography-MS (LC-MS) to analyse GLSs in leaf extracts. Among the seven classes of GLSs in the wild-type rosettes, 4-methylsulfinylbutyl glucosinolate (4MSOB) was the major component amounting to 50% of the total (Supplementary Fig. 16), consistent with a previous report^49^. When the aerial parts were analysed, total GLSs were more abundant in old plants than in young plants (Supplementary Table 1). To minimize the impact of leaf conditions we used rapidly expanding leaves (Fig. 1a) to detect the GLSs, and the results were consistent: the content of total GLSs gradually increased with plant age and the difference between the young and the adult plants was significant (Fig. 7a and Supplementary Table 2).

The cytochrome P450 monooxygenases CYP79B2 and CYP79B3 are key enzymes in the biosynthesis of indole related GLSs^50^,^51^. In *cyp79b2 cyp79b3* double mutant the aliphatic GLS content was largely unaffected but the amount of indole related GLSs was greatly reduced (Supplementary Fig. 17a,b and Supplementary Table 3), as reported^51^. However, probably because the indole-type GLSs constitute a very low proportion of total leaf GLSs, the *cyp79b2 cyp79b3* double mutant had only a mild effect on *H. armigera* larvae growth (Supplementary Fig. 17c).

Two R2R3-MYB transcription factors, MYB28 and MYB29, were shown to regulate aliphatic GLS biosynthesis^52^ and in the *myb28 myb29* double mutant aliphatic GLS were barely detectable^43^. We found that, despite a great reduction in this double mutant, the amount of residual GLSs still increased with age (Fig. 7a, Supplementary Table 2). Feeding assays showed that bollworm larvae grew much faster on *myb28 myb29* mutant leaves than on wild-type leaves, consistent with the report that generalist herbivores are typically sensitive to GLSs^53^. For the wild-type, larvae fed young plants showed a higher weight gain than those fed old plants; whereas for the *myb28 myb29* mutant, although the larvae fed on the young plants also grew faster, the difference in insect resistance between young and old plants became smaller (Fig. 7b).

We then examined the effects of miR156-targeted SPLs on the age-dependent accumulation of GLSs. In both *35S:MIR156* and *SPL9:rSPL9* leaves, the amount of GLSs was low in 20-day-old plant and high in 40-day-old plant, similar to the pattern observed in the wild-type plant (Fig. 7c and Supplementary Table 4), indicating that the enhanced GLS accumulation in old plant is probably not governed by the miR156-SPL module.

Biosynthesis of GLSs can be induced by JA or wounding^54^,^55^,^56^. Accordingly, the level of GLSs was obviously low in *coi1-2* compared with that in wild-type plants; however, in *coi1-2* plant GLSs also mounted with age (Fig. 7c and Supplementary Table 4), thus the tendency of this age-related accumulation was not impaired in this JA signalling mutant. Insect feeding assay showed that old plants exhibited enhanced resistance to bollworm larvae than young plants in all genotypes tested (Fig. 7d), and there was a clear correlation (*R*^2^=0.71, Pearson's correlation) between the insect resistance and the GLS content (Fig. 7e). These results suggest that, despite the declining JA response, other defense mechanisms, such as the constitutive accumulation of GLSs exemplified here, contribute to insect resistance in adult plants.

To test if biosynthesis of GLSs was upregulated in adult plants, we analysed the expression of five P450 genes (*CYP79B2*, *CYP79F1*, *CYP79F2*, *CYP83A1* and *CYP83B1*) in the biosynthetic pathway^57^,^58^,^59^. In contrast to the higher GLS content in old plants, transcripts of these P450s were less abundant in 40-day-old than in 20-day-old plants, regardless of the genetic backgrounds tested (Supplementary Fig. 18). This suggests that the elevated storage of GLSs in old plant leaves is unlikely due to *de novo* biosynthesis. It has been reported that GLS contents in leaves decrease to a very low level during senescence^49^. Our LC-MS analysis confirmed this dynamics: the GLSs in the first two leaves were high at the early stage and dropped notably at the senescence stage (Fig. 7f and Supplementary Table 5). Contrary again to the decline of GLSs, expression levels of the five P450s were increasing rather than decreasing in these two leaves during this period of leaf growth (Supplementary Fig. 19). Thus in senescent leaves, while biosynthesis of GLSs was still active, product storage was diminished.

At reproductive stage GLSs are transported from leaves to seeds^60^. We wondered whether GLSs could also be transported from senescent leaves to new leaves at the vegetative stage. To test this possibility, we detached the first five leaves (leaf #1–5) from 20-day-old plants to avoid or reduce GLSs export. Four days later the GLSs in the rest four leaves (leaf #6–9) were quantified. Because leaf detachment triggers a wounding response, we used the plants with wounding treatment on the first five leaves as a control, in addition to completely untreated plants as another control (Fig. 7g,h). Indeed, removal of the first five leaves reduced the accumulation of GLSs in the rest four leaves: the content was significantly lower than that in the wounding control and significantly lower than that in the intact control (Fig. 7h and Supplementary Table 6). Expression levels of the five P450 genes in wounded and cut plants either remained unchanged or elevated from the intact plant (Supplementary Fig. 20), likely due to wounding induction^55^. Although further investigation is needed, these results suggest that GLSs in senescent leaves are possibly recycled and delivered into new leaves at the later vegetative stage to augment the storage of defense compounds.

## Discussion

**Figure 8:**
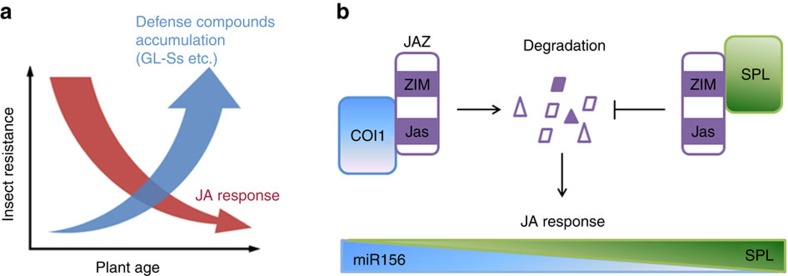
A model for the age-dependent change of plant resistance to insect herbivores. (**a**) During plant growth, the JA-regulated defense is highly active at juvenile stage but declines with age, while defense compounds accumulate constitutively, which supply the insect resistance continuously during plant growth. (**b**) The miR156-regulated SPL9 group proteins stabilize JAZs though direct binding to its N-terminal. The increased amount of SPL proteins in aged plant promotes JAZ accumulation, which in turn attenuates JA response.

Plants encounter attacks from varied populations of insect herbivores at different developmental stages. In this investigation, we report an age-related temporal change of JA response, which is highly active in the early stage of plant life and declines along with plant growth. In contrast to this tendency, defense compounds like GLSs accumulate cumulatively, contributing to the insect resistance in adult plants (Fig. 8a). Fast turn-over of JAZ proteins holds the key to JA signal output^3^,^4^, and our data suggests that SPL9 can stabilize JAZ3, possibly through protein–protein interaction. We propose that the increased amount of SPL proteins in adult plants results in elevated accumulation of JAZ proteins, which in turn dampen JA responses (Fig. 8b). In human the miR181-DUSP6 module is responsible for desensitization of T cell receptor-triggered immune signalling cascade^61^. Our finding that the miR156-targeted SPLs regulate age-dependent decline of JA-induced defense in plant resembles this miR181-a/6 (DUSP6)-mediated immunosenescence in human.

We found that DELLA proteins, which repress GA signalling, do not impair the general tendency of the age-related JA response deterioration. However, DELLAs do interact with JAZ and this interaction prevents JAZ from suppression of JA-responsive genes^33^. Here we demonstrate that SPL9 stabilizes JAZ, likely by preventing degradation through the COI1-mediated pathway which we propose results in higher JAZ activities that repress JA responses.

We propose that in the early stages after germination, when plants have little biomass and potentially an insufficient amount of defense compounds, the active JA response is required to endure or resist herbivores and possibly other pathogens. As defense metabolites accumulate with age, they may provide a higher level of basal or constitutive resistance, and thus may alleviate the burden on JA-mediated active defense. While providing plant with high resistance to herbivores and certain types of pathogens, JA inhibits plant growth by interfering with auxin and GA signals^33^,^45^,^62^. The age-dependent decay of JA signal could be a strategy of plant to ensure successful development, in which the SPL proteins contribute to the balance between defense and the growth.

Various secondary metabolites act as defense compounds to protect plants from herbivores and pathogens^63^. Interestingly, both induced and constitutive defenses may utilize the same defensive compounds. For example, gossypol and related sesquiterpene aldehydes are constitutively synthesized and stored in cotton plants, but their production is also induced by elicitation^64^. Although JA did not affect the tendency of increasing accumulation of GLSs in aging plants, it does play a role in promoting the biosynthesis of GLSs, as shown here (Fig. 7c) and reported previously^54^,^55^,^56^,^65^.

Remobilization of nutrients such as potassium, phosphorus and nitrogen from leaf to leaf during the vegetative stage or from leaves to seeds during the reproductive stage is common in plants^66^. Besides nutrients, plants also redistribute secondary compounds. In *Arabidopsis* GLSs can be transported from leaf to seed, and the nitrate transporters of GTR1 and GTR2 are responsible for this translocation^60^. In this investigation, the possibility of GLSs translocation from senescent to new leaves reflects a conceivable strategy of plant to save energy through re-use of previously synthesized defense metabolites (rather than discard them being discarded as waste). Export of substances from senescent leaves may contribute to plant constitutive defense. Finally, although GLSs play a predominant role in defense against generalist herbivores in *Arabidopsis*, the resistance to *P. xylostella*, a specialist insensitive to GLSs^65^, still increased with plant age. Besides GLSs, changes of other secondary metabolites, nutritional components and structural or mechanical factors may also augment insect resistance and compensate for decayed JA response in adult plant, which deserve further investigation.

## Methods

### Plant materials and treatments

Plants of *Arabidopsis thaliana* (ecotype Col-0 or Ler-0) were grown at 22 °C in long-day (LD, 16 h light/8 h dark) or short-day (SD, 8 h light/16 h dark) conditions, as indicated. *35S:MIR156*, *35S:MIM156*, *SPL9:rSPL9*, *SPL9:rSPL9-GR*, *coi1-2*, *myb28 myb29*, *cyp79b2 cyp79b3*, *della* and *35S:RGAδ17* are as described^20^,^43^,^47^,^51^,^67^,^68^,^69^.

For *SPL9:GFP-SPL9*, *rSPL9* (resistant to miR156) in *SPL9:rSPL9* (ref. 20) was replaced with *SPL9*. In brief, *GFP* was fused to *SPL9* coding region at 5′-terminal and expressed under *SPL9* promoter. For overexpression of miR156 and *SPL9* in *coi1-2*, the constructs of *35S:MIR156* and *SPL9:rSPL9* (ref. 23) were used. For *JAZ3:JAZ3-FLA*G, *JAZ3* promoter and the coding region linked to FLAG were cloned into pCAMBIA1300. For overexpression of JAZ3-HA, JAZ3δC-HA and JAZ3δN-HA in *Arabidopsis*, the coding regions of *JAZ3*, *JAZ3δC* and *JAZ3δN* were inserted into the JW819 vector^29^ behind the 35 S promoter, respectively, with a 3 × HA C-terminal fusion. To obtain transgenic plants, binary vectors harbouring the desired construct were transferred into *Agrobacterium tumefaciens* strain GV3101 (pMP90) by the freeze–thaw method. Transgenic *Arabidopsis* plants were generated by a floral dip method, and screened with 0.05% glufosinate (Basta) on soil, 40 μg ml^−1^ hygromycin or 50 μg ml^−1^ kanamycin on half-strength MS plates.

As shown in Fig. 1a and Supplementary Fig. 1a, when grown in LD the ∼14-day-old and the ∼26-day-old plants were considered young and old (or adult), and when in SD the ∼20-day-old and the ∼40-day-old plants were considered young and old, respectively. Leaves were harvested from plants at an indicated plant age (day post germination, DPG). The new leaves used for analysis of hormone responsive genes were ∼3 mm in width (Fig. 1a, black arrows). The rapidly expanding leaves (Fig. 1a, red arrows), or the total aerial tissues were used for insect feeding test, wounding treatment and GLS analysis, as indicated in the legend to the figure. And the sampling time could be adjusted according to leaf growth stage, as indicated.

For hormone treatments, MeJA (Aldrich), GA_3_ or PAC (Sigma-Aldrich) was dissolved in ethanol to 50 mM and added to double-distilled water with the final concentration of 50 μM. Water solution with an equal volume of ethanol was used as mock. The hormone solution was sprayed to aerial parts of the tested plants. For MeJA treatments, samples were collected at an indicated time for analysis. For GA_3_ and PAC treatments, ∼10 days after germination plants were sprayed one time per day. For wounding treatments, rapidly expanding leaves at an indicated DPG were punched (approximately one-third area of the leaf were punched with a 10 μl pipette tip) and harvested two hours post treatment, as indicated.

For transient induction of SPL expression in *SPL9:rSPL9-GR* plant, DEX (Sigma-Aldrich) was dissolved in ethanol to 10 mM and added to double-distilled water with the final concentration of 10 μM. Water solution with an equal volume of ethanol was used as mock. The solutions were sprayed to aerial parts of the 12-day-old plants; 12 h later, the DEX pretreated plants were sprayed with 50 μM MeJA and the leaves of the first pair were harvested 4 h post treatment.

### Insect culture and feeding test

Eggs of cotton bollworm (*Helicoverpa armigera*) and Diamondback moth (*Plutella xylostella*) were obtained from Nanjing Agricultural University. The larvae were reared in the laboratory at 25 °C, 70% relative humidity and a 14-h-light/10-h-dark cycle on a modified artificial diet, as described^35^. For each feeding experiment, synchronous third instar larvae of *H. armigera* and second instar larvae of *P. xylostella* were selected, weighed individually and divided into groups with each group containing 25–30 individuals. For whole plant feeding, each pot of the plants contained five individuals of *H. armigera* larvae or 10 individuals of *P. xylostella* larvae, and the pot was capsulated with plastic wrap to contain the larvae. For leaf feeding, the detached leaves and an individual larva were placed in a container. After feeding for 3 days, net weight increases were recorded.

### Gene expression analyses

Total RNAs were isolated from *Arabidopsis* plants by Trizol reagent (Invitrogen). Total RNA (1 μg) was treated with 1 μl of DNase I (1 unit per ml; Fermentas) and used for preparing the first strand cDNA (Invitrogen). qRT-PCR was performed and S18 (At4g09800) was used as internal standard. Biological triplicates with technical duplicates were performed. All nucleotide primers used in this investigation are listed in Supplementary Table 7.

### Yeast two- and three-hybrid assay

For yeast two-hybrid assay, a series of *SPL*s was introduced into the pGBKT7 (Clontech), as described^32^, and *COI1*, *JAZ1*, *JAZ2*, *JAZ4*, *JAZ6*, *JAZ7*, J*AZ9*, *JAZ10*, J*AZ11*, *JAZ12* and *JAZ3* (*JAZ3*, *JAZ3δC* and *JAZ3δN*) were inserted into pGADT7 (Clontech), respectively. The *MYC2*/pGADT7 construct was as described^34^. Plasmids were transferred into yeast strain AH109 (Clontech) by the LiCl-polyethylene glycol method. Transformants were selected on SD-Leu-Trp plates. The interactions were tested on SD-Leu-Trp-His or SD-Ade-Leu-Trp-His plates with 3-amino-1,2,4-triazole, incubating for 3–4 days at 30 °C. At least 10 individual clones were analysed. For yeast three-hybrid assay, JAZ3 and SPL9 were inserted into pBridge (Clontech), forming a *JAZ3-SPL9*/pBridge construct. The yeast strain AH109 was co-transformed with a *JAZ3-SPL9*/pBridge and a *COI1*/*pGADT7* construct and plated on SD-Leu-Trp selective dropout medium. Colonies were transferred to the appropriate selective dropout liquid medium (SD-Leu-Trp-His) with 40 μM coronatine (Sigma-Aldrich) and different concentrations of methionine. SPL9 expression from the pBridge construct was controlled by the P_Met25_ promoter, and the SPL9 level was decreasing along with the increasing concentrations of Met.

### Pull-down and immunoblot analyses

Proteins were extracted from leaves by an extraction buffer (50 m MHEPES, 10 mM EDTA, 50 mM NaCl, 10% glycerol, 1% polyvinylpolypyrrolidone, 2 mM DTT, 1 mM phenylmethylsulfonyl fluoride, 10 mM MG-132, and 1 × protease inhibitor cocktail, pH 7.5). Immunoblotting was performed by loading proteins onto a 10% SDS–polyacrylamide gel electrophoresis gel (80 μg proteins per lane). After electrophoresis, the proteins were electrotransferred to a Hybond-C membrane (Amersham). The 3 × HA fusion proteins were detected by immunoblot with anti-HA-peroxidase antibody (Cat No. 12013819001, Roche; dilution, 1:2,000). GFP-SPL were detected by immunoblot with GFP antibody (Cat No. 11814460001, Roche; dilution, 1:2,000). Sensitive detection of the bound antibody was performed using ECL Plus Western Blotting kit (Thermo) according to the manufacturer's protocol. The full view of cropped blots is given in Supplementary Fig. 21.

For expression of HIS-SPL9, the *SPL9* fragment was inserted into pET32a (Stratagene) between the cloning sites of *Bam*HI and *Sac*I. For purification of HIS-SPL9, the recombinant protein was expressed in *Escherichia coli* strain BL21 (DE3). Total proteins of *E. coli* were extracted by lysis buffer containing 50 mM Tris-Cl, pH 7.6, 100 mM NaCl, 25 mM imidazole, 10% (v/v) glycerol, 0.1% (v/v) Tween 20, 1 mM phenylmethanesulfonyl fluoride (PMSF), 10 uM MG132 (Sigma-Aldrich), and Protease Inhibitor Cocktail (Roche). Insoluble debris was removed by centrifugation at 13,000*g* for 10 min at 4 °C and the supernatant was applied to a Ni affinity column (Ni-NTA resin, Qiagen), which was then washed with 3 × volumes of lysis buffer, and finally eluted with lysis buffer that contained 250 mM imidazole. Agrobacteria-infiltrated tobacco (*Nicotiana benthamiana*) leaves were used for expressing truncated JAZ3 proteins with 3 × HA tag fused at C-terminal. For immunoprecipitation, total tobacco proteins containing either JAZ3δN-HA or JAZ3δC-HA were mixed with 80 μg recombinant HIS-SPL9 in 1 ml. Ni-NTA resin (Qiagen) was used to bind HIS-SPL9. After incubation for one hour at 4 °C, the Ni-NTA resin was washed and eluted with imidazole. Samples were used to detect the truncated fusion proteins of JAZ3-HA by immunoblotting using anti-HA, meanwhile a portion of the samples was used for Coomassie Brilliant Blue staining to detect the amount of HIS-SPL9.

### Analysis of JA and JA-Ile concentrations in plant tissues

The detection was performed using a modified protocol as described^70^. About 0.2 g leaf samples were homogenized and added with 1 ml ethyl acetate. Following centrifugation at 13,000 r.p.m. (16,100*g*) for 10 min at 4 °C, the supernatant was transferred to a 2-ml tube; the precipitation was extracted with additional 1 ml ethyl acetate. The supernatants were combined and evaporated to dryness on a vacuum concentrator. Residue of each sample was resuspended in 200 μl of 70% methanol (v/v) and centrifuged, and the supernatant was used for JA and JA-Ile detection.

Samples were analysed by reversed-phase chromatography on an Waters Ultra Performance Liquid Chromatography (UPLC), using a 1.7 μm, 2.1 × 50 mm BEH C18 column (Waters). Water with 0.05% formic acid (A) and methanol (B) were used as the mobile phase. The gradient program was: 0–1 min, 15% B; 1–8 min, 15–98% B; 8–9 min, 98% B; 9–9.1 min, 98–15% B; 9.1–13 min, 15% B, with a flow rate of 0.3 ml min^−1^. A coupled Thermo triple-quadrupol-MS HESI mass spectrometer (TSQ quantum access max) was used to collect MS data in negative ion mode. The parameters of MS was set as fellow: spray voltage: 2800; vaporizer temperature: 320; sheath gas pressure: 35; aux gas pressure: 10; capillary temperature: 350. The phytohormones were identified by comparing the key ion fragmentation in SRM scan mode, with the parameters: Q1FM: 209.000; Q1FM: 59.000; Q1PW: 0.70; Q3PW: 0.70; collision energy: 15; tube lens: 70 (JA). Q1FM: 322.000; Q1FM: 130.000; Q1PW: 0.70; Q3PW: 0.70; collision energy: 22; tube lens: 70 (JA-Ile). For JA, the authentic standard was used for confirmation.

### Glucosinolates extraction and detection

GLS were analysed as described^43^,^49^. Plant materials, immediately after harvest, were frozen in liquid nitrogen and lyophilized to dryness. The lyophilized tissue (10–20 mg) was extracted by 70% methanol (3 ml) containing 0.05–0.1 μmol of an internal standard (sinigrin) and incubated under 60 °C for 1 h at shaking. Samples were then cooled to 4 °C and centrifuged at 4000, g. The supernatant was loaded onto an anion-exchange column (DEAE-Sephadex A-25). After washing with 4 ml sodium acetate (20 mM) the column was capped and treated overnight with 10 μl of arylsulfatase (Helix Pomatia Type H-1, Sigma) to convert the GLS to their desulfated derivatives, which were eluted from the column with double-distilled water.

Samples were separated on a 6120 Quadrupole LC-MS system (Agilent) fitted with a C-18 reversed-phase column (syncronis RP-18, 250 × 4.6 mm i.d., 5 um particle size, Thermo Scientific), using a water (Solvent A)-acetonitrile (Solvent B) gradient at a flow rate of 1 ml min^−1^. The 52 min run at ambient room temperature consisted of 1.5% B (1 min), 1.5–5.0% B (5 min), 5.0–7.0% B (2 min), 7.0–21.0% B (10 min), 21.0–29.0% B (5 min), 29.0–43.0% B (7 min), 43.0–93.0% B (0.5 min), a 4-min hold at 93.0% B, 93.0–1.5% B (0.5 min) and a 7-min hold at 1.5% B. The following parameters were used to obtain positive ionisation: dry gas flow at 12.0 l min^−1^, temperature 350 °C, mass range 100–1500, fragmentor 70, threshold 150. Eluent was monitored by diode array detection between 190 and 60 nm (2 nm interval). Desulfated GLS were identified by comparison of retention time and MS data to those previously reported, and quantified by A229 nm relative to the internal standard.

### Data availability

The authors declare that all data supporting the findings of this study are available within the article and its Supplementary Information files or are available from the corresponding author upon request.

## Additional information

**How to cite this article:** Mao, Y.-B. *et al*. Jasmonate response decay and defense metabolite accumulation contributes to age-regulated dynamics of plant insect resistance. *Nat. Commun.* **8,** 13925 doi: 10.1038/ncomms13925 (2017).

**Publisher's note:** Springer Nature remains neutral with regard to jurisdictional claims in published maps and institutional affiliations.

## Supplementary Material

Supplementary Information Supplementary Figures and Supplementary Tables
