# Supplementary material for: Jasmonate response decay and defense metabolite accumulation contributes to age-regulated dynamics of plant insect resistance
Source: Nat Commun. 2017 Jan 9;8:13925. doi: 10.1038/ncomms13925 (PMC5233801; doi:10.1038/ncomms13925)
Supplement: Supplementary Information — Supplementary Figures and Supplementary Tables [file ncomms13925-s1.pdf]

Supplementary Figures

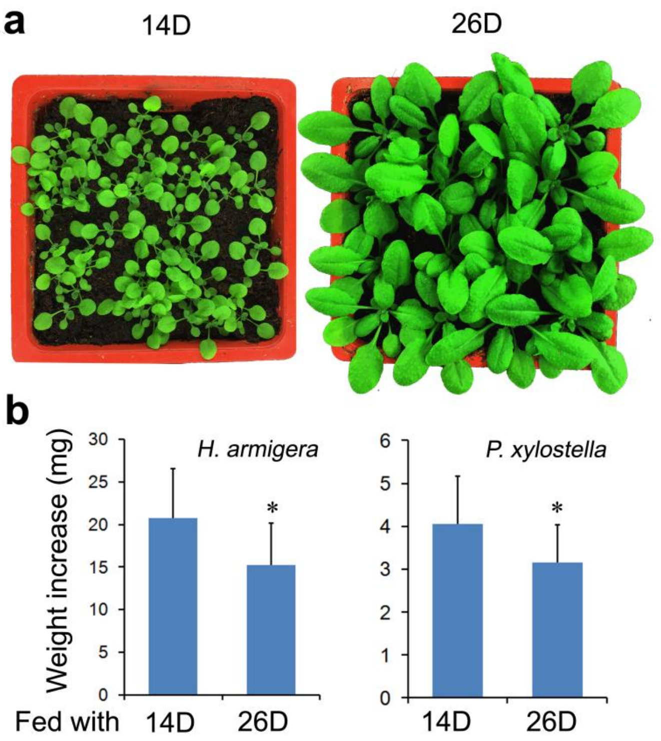

Supplementary Figure 1. Old plants are more resistant to insect herbivores than young plants. (**a**) Image of young (14-day-old, 14D) and old (26-day-old, 26D) plants of *Arabidopsis thaliana* (Col-0) grown in LD. (**b**) Weight increase of *H. armigera* and *P. xylostella* larvae. 3rd instar larvae of *H. armigera* and 2nd instar larvae of *P. xylostella* were fed with the whole plants of 14D and 26D as shown in (**a**), respectively, for 3 days. Each pot of the plants contained 5 individuals of *H. armigera* larvae or 10 individuals of *P. xylostella* larvae and the pot was capsulated with plastic wrap to make sure that the larvae were confined. Data are shown as mean ±s.d. (n=25), asterisk indicates significant difference from the 14D group (Student's *t*-test, *p<0.05).

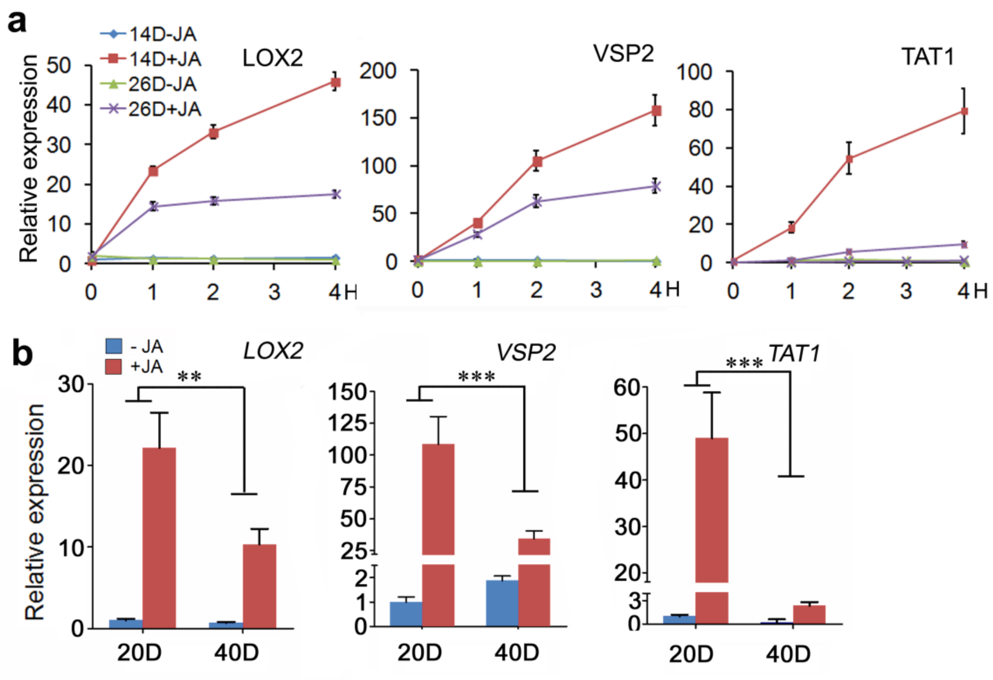

Supplementary Figure 2. JA response attenuated with age both in plants under LD and SD condition. Error bars represent ±s.d. (n=3). Data were analyzed by multiple comparisons (Tukey test) followed by two-way ANOVA (*p<0.05, **p≤0.01, ***p≤0.001).

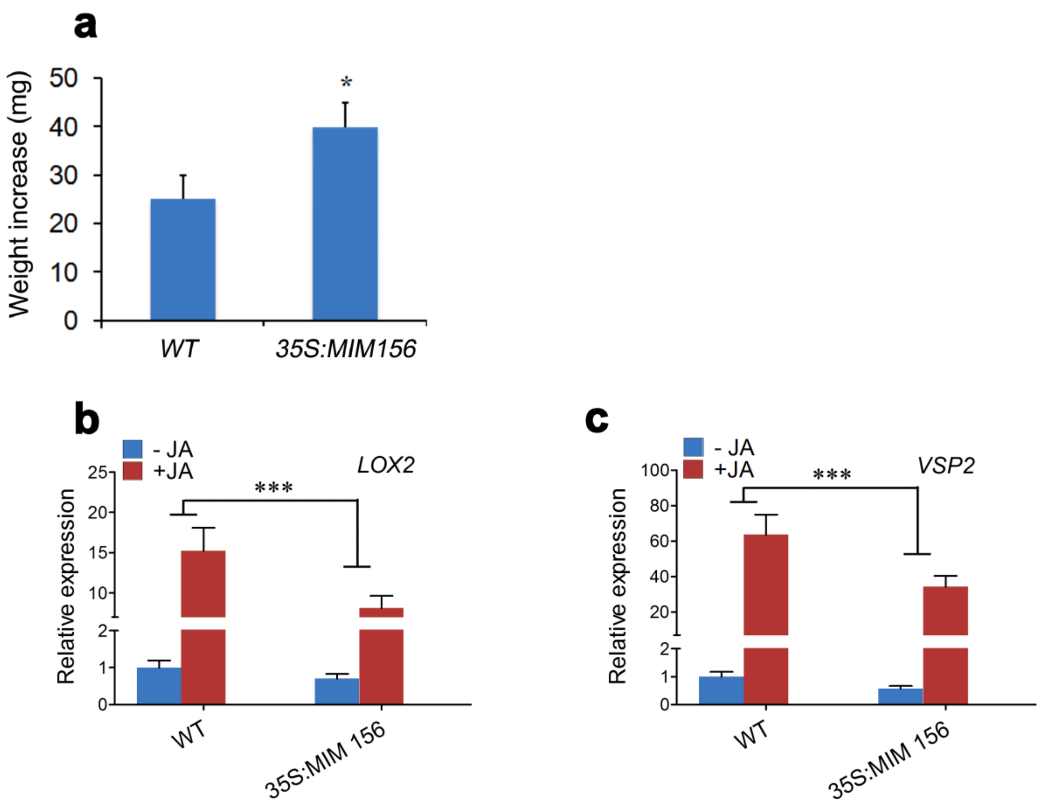

Supplementary Figure 3. Blocking miR156 with *Pro35S:MIM156* attenuates JA response and insect resistance. (**a**) Weight increase of cotton bollworm (*Helicoverpa armigera*) larvae fed with leaves of the wild type (WT) or the *Pro35S:MIM156* plants grown in SD. Leaves from each plant (30D in SD) were used to feed the 3rd-instar larvae, weight increase was recorded three days later. Data are shown as mean ±s.d. (n=25), asterisk indicates significant difference from the WT group (Student's *t*-test, $p<0.05$). (**b**, **c**) Expression of *LOX2* and *VSP2* in the WT and the *35S:MIM156* plants four hours after MeJA treatment. The 30-day-old plants in SD were treated with 50 μM MeJA (+JA) or ethanol as control (-JA). New leaves with ~3 mm in width were harvested for qRT-PCR. Plants were grown in SD because the *Pro35S:MIM156* plants were used in the analysis. Data were analyzed by multiple comparisons (Tukey test) followed by two-way ANOVA (***$p\leq0.001$). Error bars represent ±s.d. (n=3).

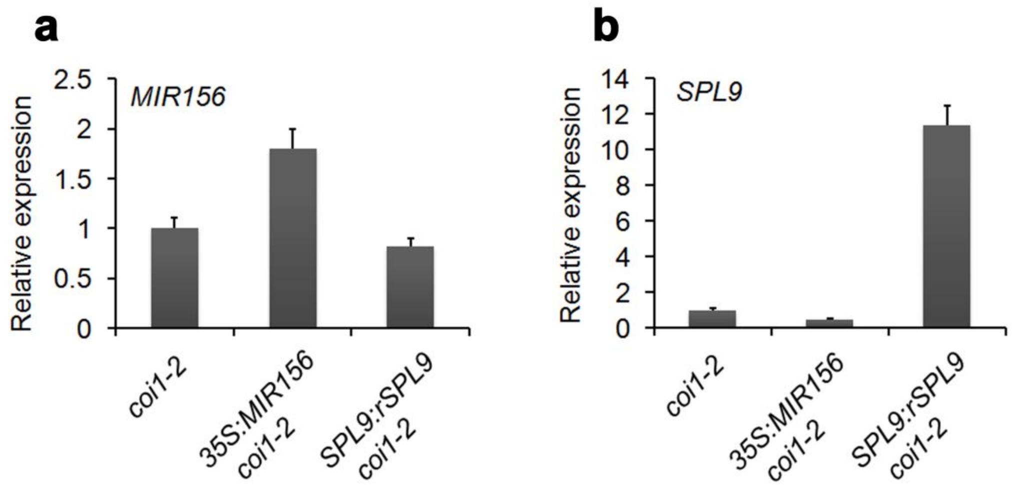

Supplementary Figure 4. Examination of *35S:MIR156* and *SPL9:rSPL9* transgene expression. One-week-old seedlings of *coi1-2*, *35S:MIR156 coi1-2* and *SPL9:rSPL9 coi1-2* were harvested and subjected to qRT-PCR to detect transcripts of pri-*MIR156b* (**a**) and *SPL9* (**b**). The expression in *coi1-2* was set to 1, error bars represent ±s.d. (n = 3).

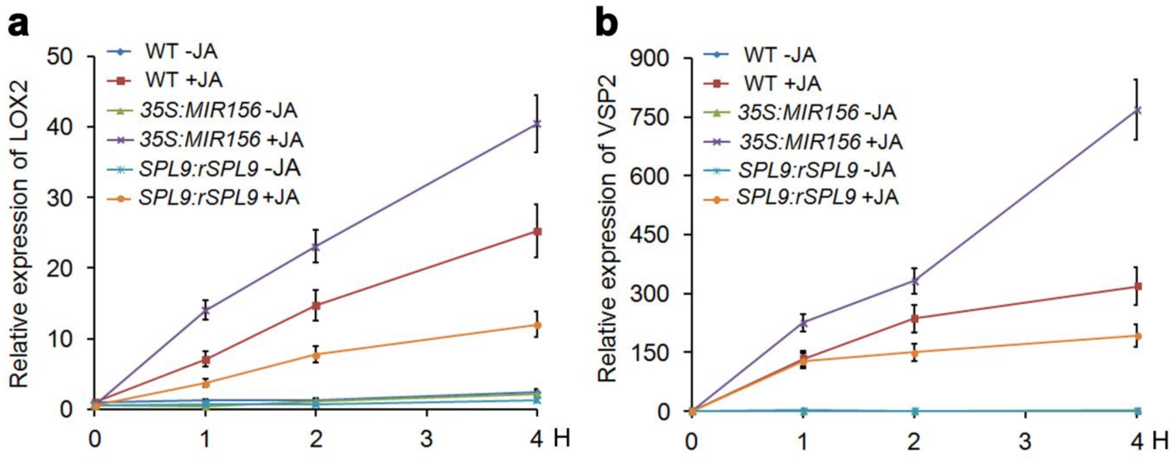

Supplementary Figure 5. Effects of *35S:MIR156* and *SPL9:rSPL9* on JA response. Expression levels of *LOX2* (**a**) and *VSP2* (**b**) in leaves at different times (hours, H) post-MeJA treatment. Plants were treated with 50 μM MeJA (+JA) or ethanol (-JA) as control. The first pair of leaves (~3 mm in width) were collected from plants of the indicated genotypes, and gene expressions were analyzed by qRT-PCR. Error bars represent ±s.d. (n=3). The plants were grown in SD because the SPL9-overexpression plants (*SPL9:rSPL9*) were included in the comparative analysis. Note that the JA response was high in *35S:MIR156* plants and reduced in *SPL9:rSPL9* plants.

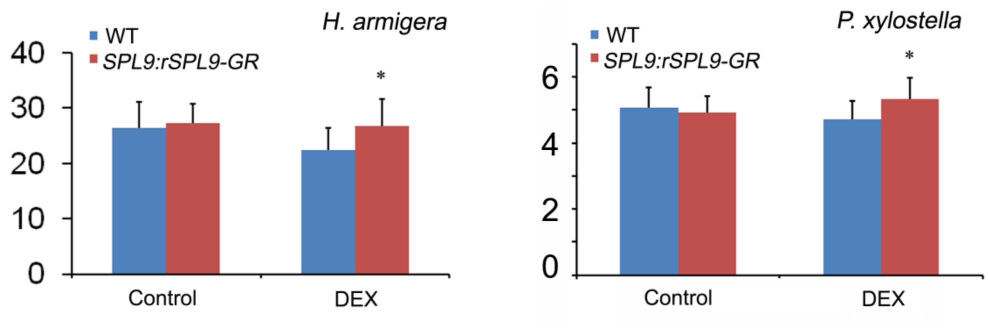

Supplementary Figure 6. Translocation of SPL9 into nucleus by DEX treatment attenuates insect resistance. The wild-type and *SPL9:rSPL9-GR* plants (12D in LD) were sprayed with ethanol (control) or 10 μM DEX. After 12 hours the whole plants were used to feed *H. armigera* and *P. xylostella* larvae, weight increase was recorded three days later. Data are shown as mean ±s.d. (n=25), asterisk indicates significant difference from the WT group (Student's *t*-test, $p<0.05$).

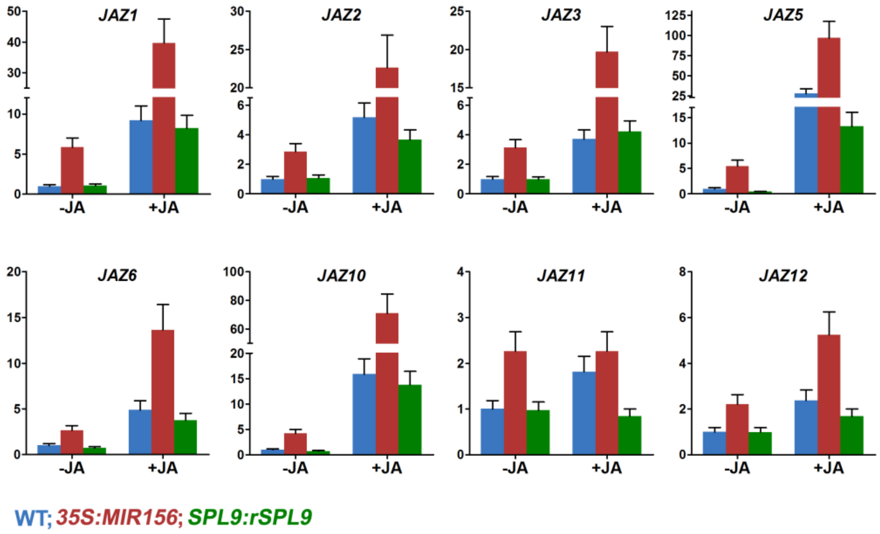

Supplementary Figure 7. Expression of *JAZ* genes in leaves of wild type (WT), *35S:MIR156* and *SPL9:rSPL9* plants in response to JA treatment. The 30-day-old plants in SD were treated with 50 μM MeJA (+JA) or ethanol (-JA) as control. New leaves with ~3 mm in width were harvested for qRT-PCR. Note that transcription of *JAZ* genes was drastically induced by JA in *35S:MIR156* plants but the induction was either not clear or reduced in *SPL9:rSPL9* plants. Error bars represent ±s.d. (n = 3).

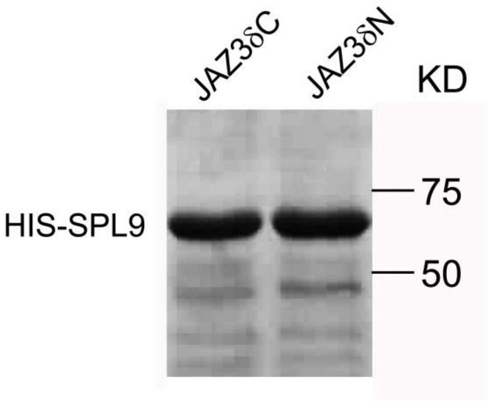

Supplementary Figure 8. Coomassie Brilliant Blue Staining of HIS-SPL9 used in Pull-down assay. The purified recombinant HIS-SPL9 was mixed with total tobacco proteins containing either JAZ3δC-HA (JAZ3δC) or JAZ3δN-HA (JAZ3δN). Ni-NTA resin (Qiagen) was used to bind HIS-SPL9. After incubation for one hour at 4°C, the Ni-NTA resin was washed and eluted with imidazole. Samples were used for immunoblot assay to detect the truncated fusion proteins of JAZ3 (Fig. 4d); meanwhile a portion of the samples was used for Coomassie Brilliant Blue staining to detect HIS-SPL9. KD, kilodalton.

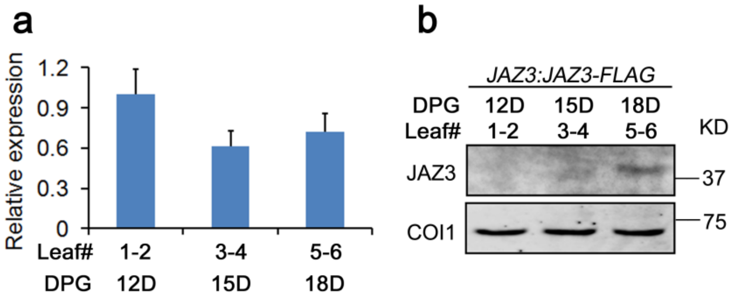

Supplementary Figure 9. Detection of JAZ3 transcript and protein levels in the first three pairs of leaves. New leaves (leaves#1-2, 3-4 and 5-6) were collected from the indicated plants at different ages (day post germination, DPG) in LD. (**a**) Transcript level in leaves of wild-type plants analyzed by RT-PCR. (**b**) JAZ3-FLAG fusion protein in leaves of *JAZ3:JAZ3-FLAG* plants detected by immunoblotting with anti-FLAG antibody. COI1 was detected using anti-COI1 antibody. KD, kilodalton. *JAZ3-FLAG* was expressed under the control of the native *JAZ3* promoter, and the fusion protein was undetectable by immunoblotting in the first two pairs of leaves, but became evident in the third pair.

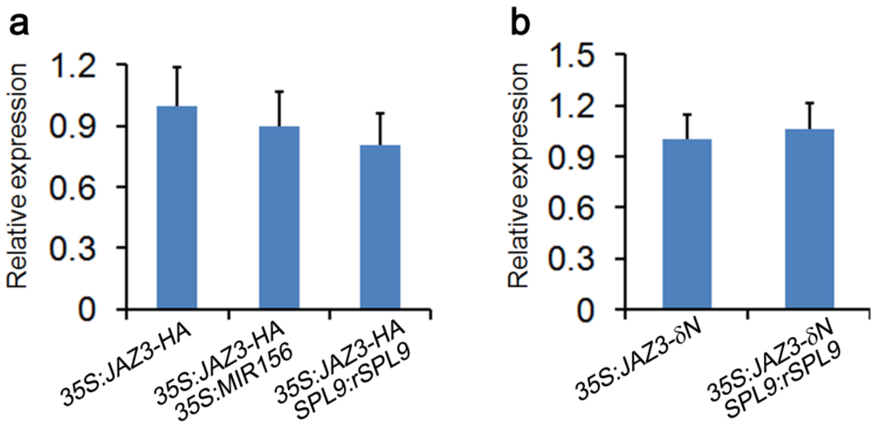

Supplementary Figure 10. Expression of *JAZ3-HA* and *JAZ3δN-HA* in the indicated transgenic plants. One-week-old seedlings were harvested; *JAZ3-HA* (**a**) and *JAZ3δN-HA* (**b**) level were analyzed by qRT-PCR. The transcript level in the wild-type background was set to 1. Error bars represent ±s.d. (n = 3). Note that both the *JAZ3-HA* and *JAZ3δN-HA* did not show a drastic difference in their transcript levels after introduction into the *35S:MIR156* or *SPL9:rSPL9* plants by crossing.

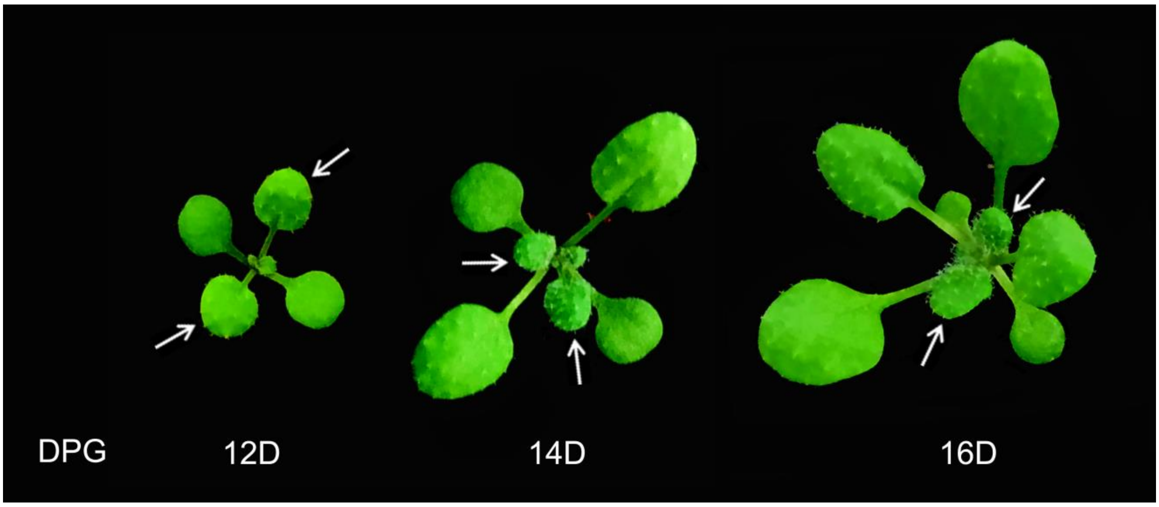

Supplementary Figure 11. Images of *35S:MIR156* plants at indicated days post-germination (DPG). White arrows indicate the leaves (leaf #1-2, 3-4 and 5-6 in plants of 12D, 14D and 16D in LD, respectively) used in Fig. 5e.

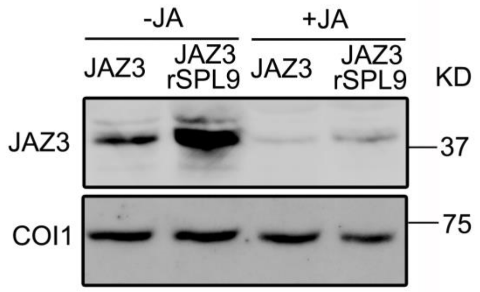

Supplementary Figure 12. JAZ3-HA protein level in *35S:JAZ3-HA* and *35S:JAZ3-HA SPL9:rSPL9* plants. Plants were treated with 50 μM MeJA (+JA) or ethanol (-JA), and the newly initiated leaves of the first pair were collected one hour later or at the indicated time post-treatment for immunoblot assay. Note that rSPL9 promoted JAZ3 accumulation. JAZ3-HA were detected using anti-HA antibody. COI1 in each sample was detected using anti-COI1 antibody. KD, kilodalton.

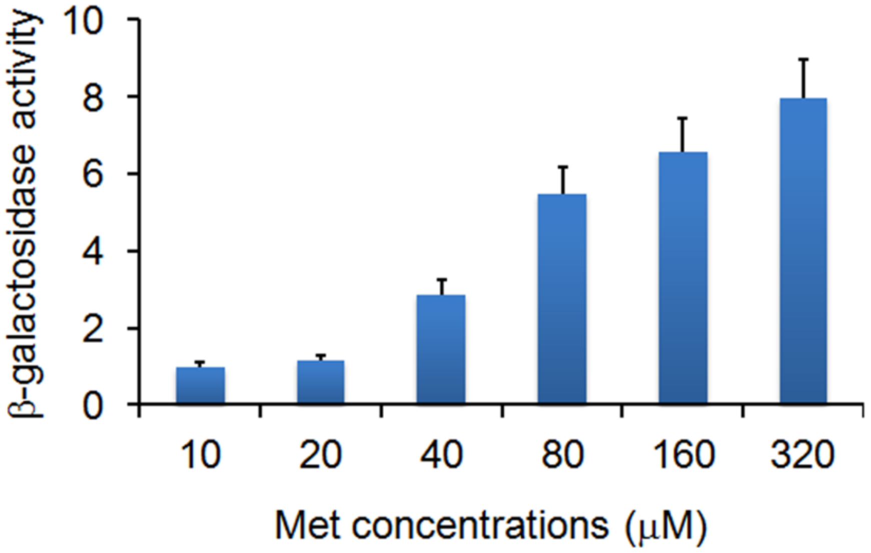

Supplementary Figure 13. Yeast three-hybrid assay of the influence of SPL9 on COI1-JAZ3 interaction. The COI1-JAZ3 binding activities are represented by β-galactosidase activity, and the promoter driving *SPL9* expression was suppressed by increasing concentrations of methionine (Met). Error bars indicate ±s.d. of three technical replicates, and the results were consistent in three biological replicates.

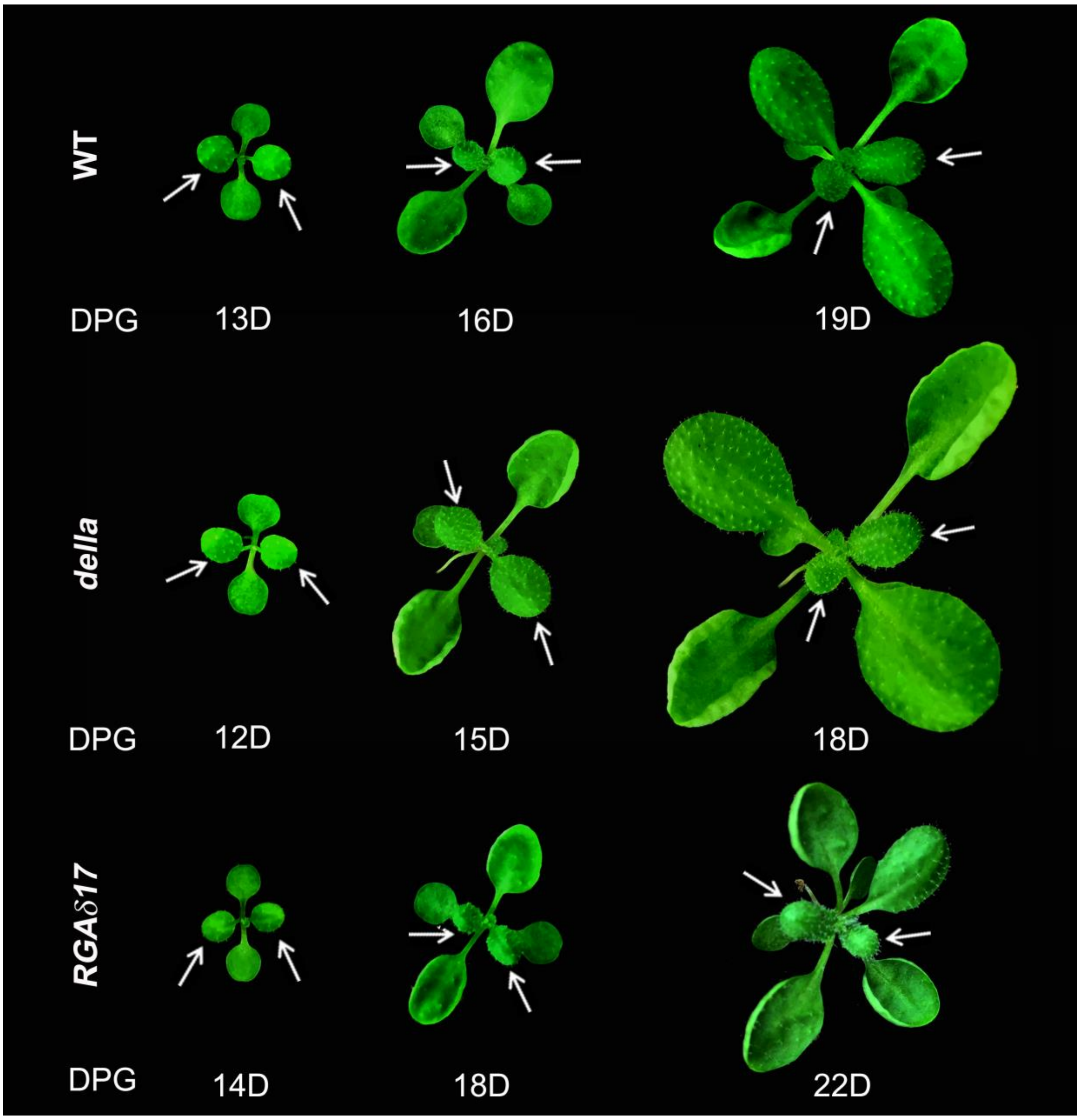

Supplementary Figure 14. Images of wild-type (WT), the penta *della*-deficient mutant (*della*) and the DELLA over-expressor (*35S:RGAδ17*) plants at indicated days post-germination (DPG). All plants were in Ler-0 background and grown in LD. White arrows indicate new leaves (leaf #1-2, 3-4 and 5-6 in plants as indicated) used in Fig. 6a-c.

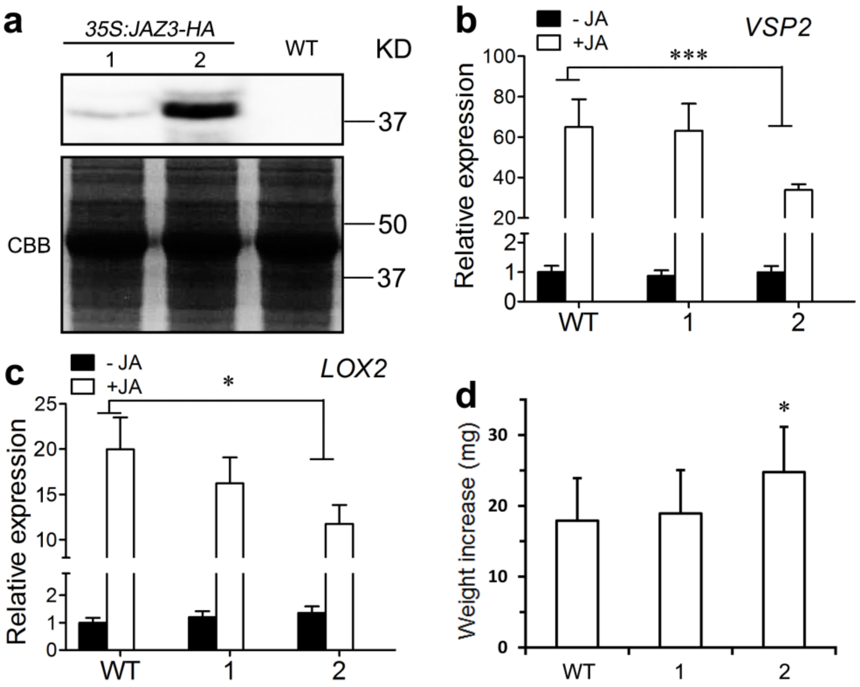

Supplementary Figure 15. High JAZ3 protein level compromises plant resistance to *Helicoverpa armigera*. (**a-c**) Examination of JAZ3 protein level and JA response in transgenic *35S:JAZ3-HA* plants. (**a**) One-week-old wild-type (WT) and *35S:JAZ3-HA* (line 1 and 2) seedlings were harvested and detected with anti-HA antibody. The amount of total proteins in each loading was monitored with Coomassie Brilliant Blue (CBB) staining. KD, kilodalton. (**b-c**) Expression of *VSP2* (**b**) and *LOX2* (**c**) in WT and *35S:JAZ3-HA* seedlings. One-week-old seedlings were treated with 50 μM MeJA (+JA) or ethanol as control (-JA), and harvested 4 hours later for qRT-PCR. The expression in the WT control plants (-JA) was set to 1. Data were analyzed by multiple comparisons (Tukey test) followed by two-way ANOVA (*$p<0.05$, ***≤0.001). Error bars represent ±s.d. (n=3). (**d**) Weight increase of 3rd instar larvae fed on leaves from the indicated plants for 3 days. Four rapidly expanding leaves from each 24D plant in LD were collected and used to feed the larvae (25-30 individuals each group). Data are shown as mean ±s.d. (n=25). Asterisk indicates significant difference from the WT group (Student's *t*-test, *$p < 0.05$).

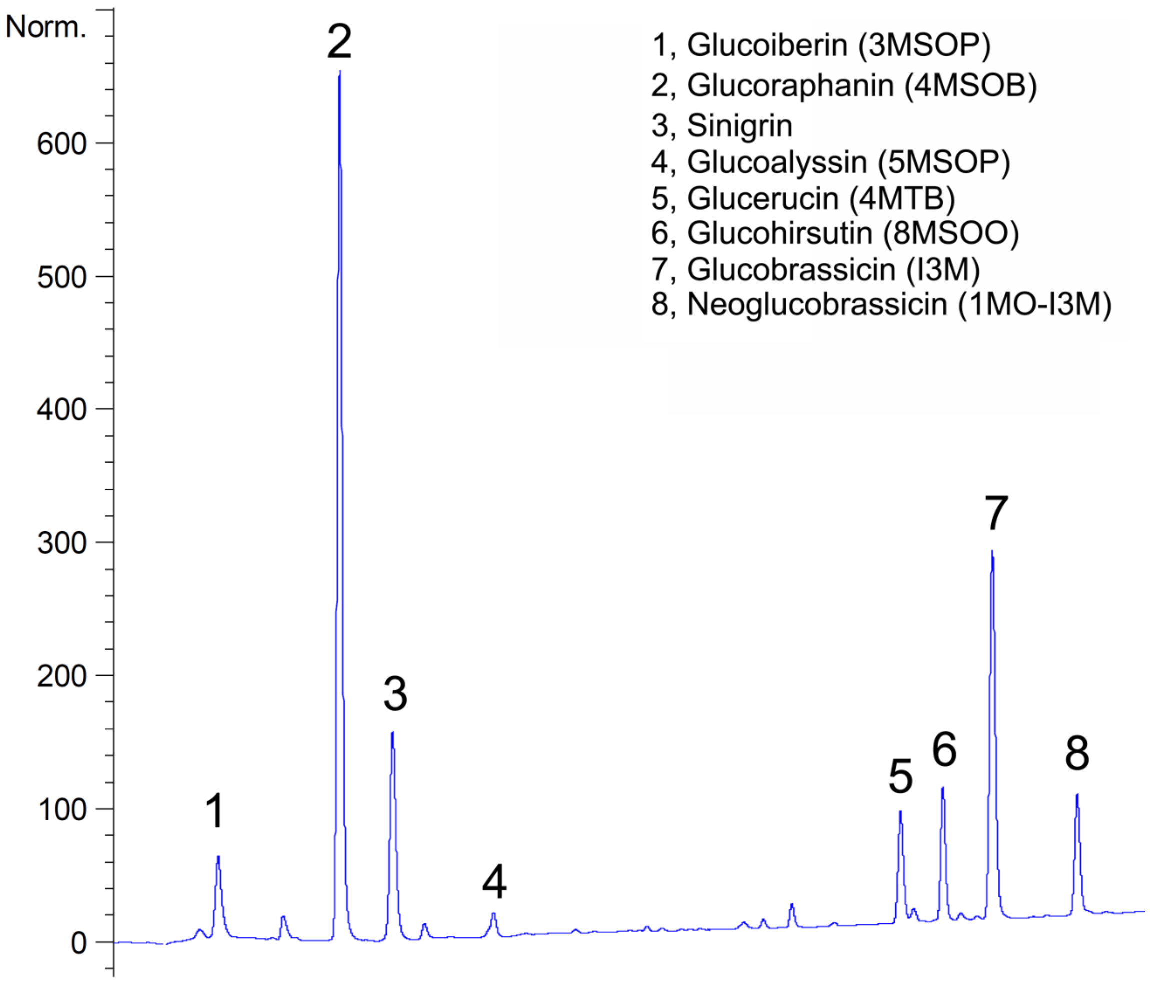

Supplementary Figure 16. LC-MS chromatogram of glucosinolates. From each of the 24-day-old wild-type plants in LD four new leaves were collected for liquid chromatography-mass spectrometry (LC-MS) analysis, and sinigrin was included as internal standard. Samples were separated on a 6120 Quadrupole LC-MS system (Agilent) fitted with a C-18 reversed-phase column, using a water (Solvent A)-acetonitrile (Solvent B) gradient at a flow rate of 1 ml min^{-1}. 1-8 indicate glucoiberin (3MSOP), glucoraphanin (4MSOB), sinigrin, glucoalyssin (5MSOP), glucerucin (4MTB), glucohirsutin (8MSOO), glucobrassicin (I3M) and neoglucobrassicin (1MO-I3M), respectively.

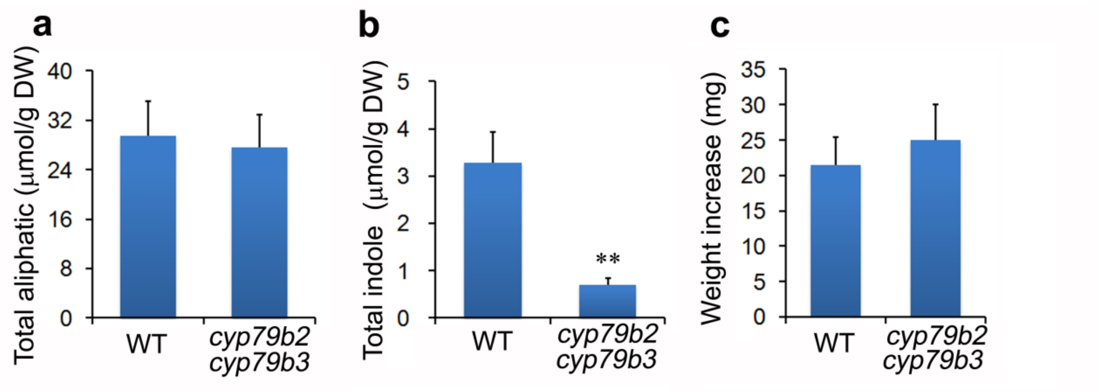

Supplementary Figure 17. Plant resistance to *H. armigera* in wild-type and *cyp79b2 cypb3*. (**a**-**b**) Total amount of aliphatic (**a**) and indole (**b**) GLSs in leaves at rapidly expanding stage from the 24D old plants (in LD) of wild-type and *cyp79b2 cyp79b3*. Data are shown as mean ±s.d. (n=3), asterisk indicates significant difference from WT (Student's *t*-test, **$p<0.01$). (**c**) Weight increase of 3rd instar larvae of *H. armigera* fed on leaves described in (**a**-**b**).

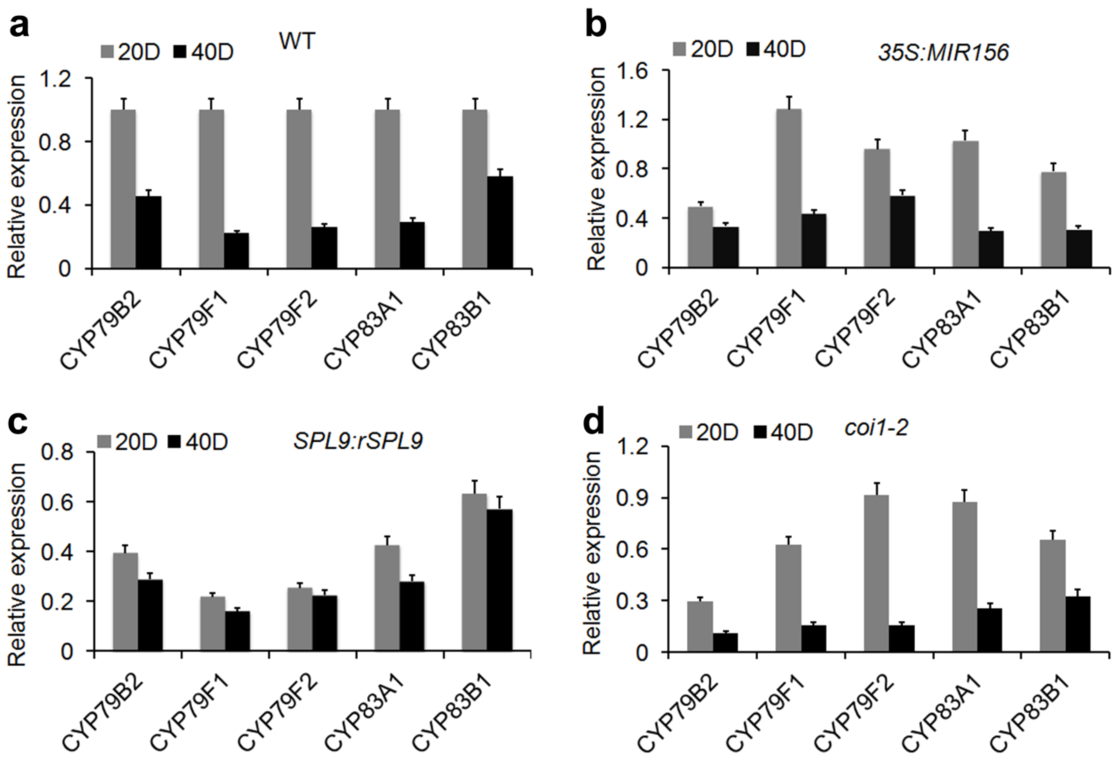

Supplementary Figure 18. Relative expression levels of P450 genes involved in biosynthesis of glucosinolates in plants of different ages. (**a-d**) The newly initiated pair of leaves from the 20D and the 40D old plants in SD of WT (**a**), *35S:MIR156* (**b**), *SPL9:rSPL9* (**c**) and *coi1-2* (**d**) were harvested for qRT-PCR. The expression of each P450 gene in the 20D WT plant was set to 1. Plants were grown in SD because the SPL9-overexpression plants (*SPL9:rSPL9*) were included in the analysis. Note that, in all these genetic backgrounds, the expression level of these P450 genes was high in young plant leaves and decreased in elder plant leaves.

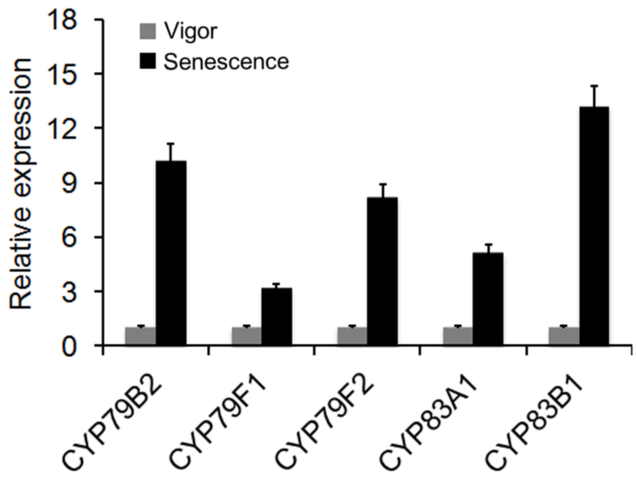

Supplementary Figure 19. Change of expression levels of the P450 genes in the first pair of leaves along with leaf maturation. The expression levels in the first two leaves at fast growing (vigorous stage, Vigor) from the 14D plants, or at senescent stage (Senescence) from the 28D plants, were compared. Plants were grown in LD and the expression of each P450 gene in Vigor was set to 1. Note that the expression level of these P450 genes was higher in the senescent leaves.

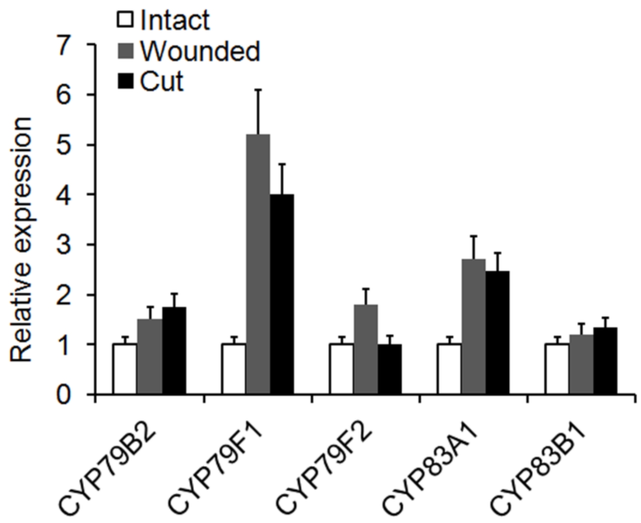

Supplementary Figure 20. Examination of relative expression levels of the P450 genes in leaves used in cutting experiments. The 1st-5th leaves were wounded (Wounded) or detached (Cut) from the 22-day-old plant grown in LD, four days later the expression of the P450 genes in the later initiated leaves (6th-9th) from Cut plants were detected, and the same set of leaves from the Wounded plants and the Intact plants were analyzed as controls. The expression of P450s in the intact plant leaves was set to 1. Error bars represent ±s.d. (n = 3). For contents of glucosinolates in these leaves, see Figure 6g-j.

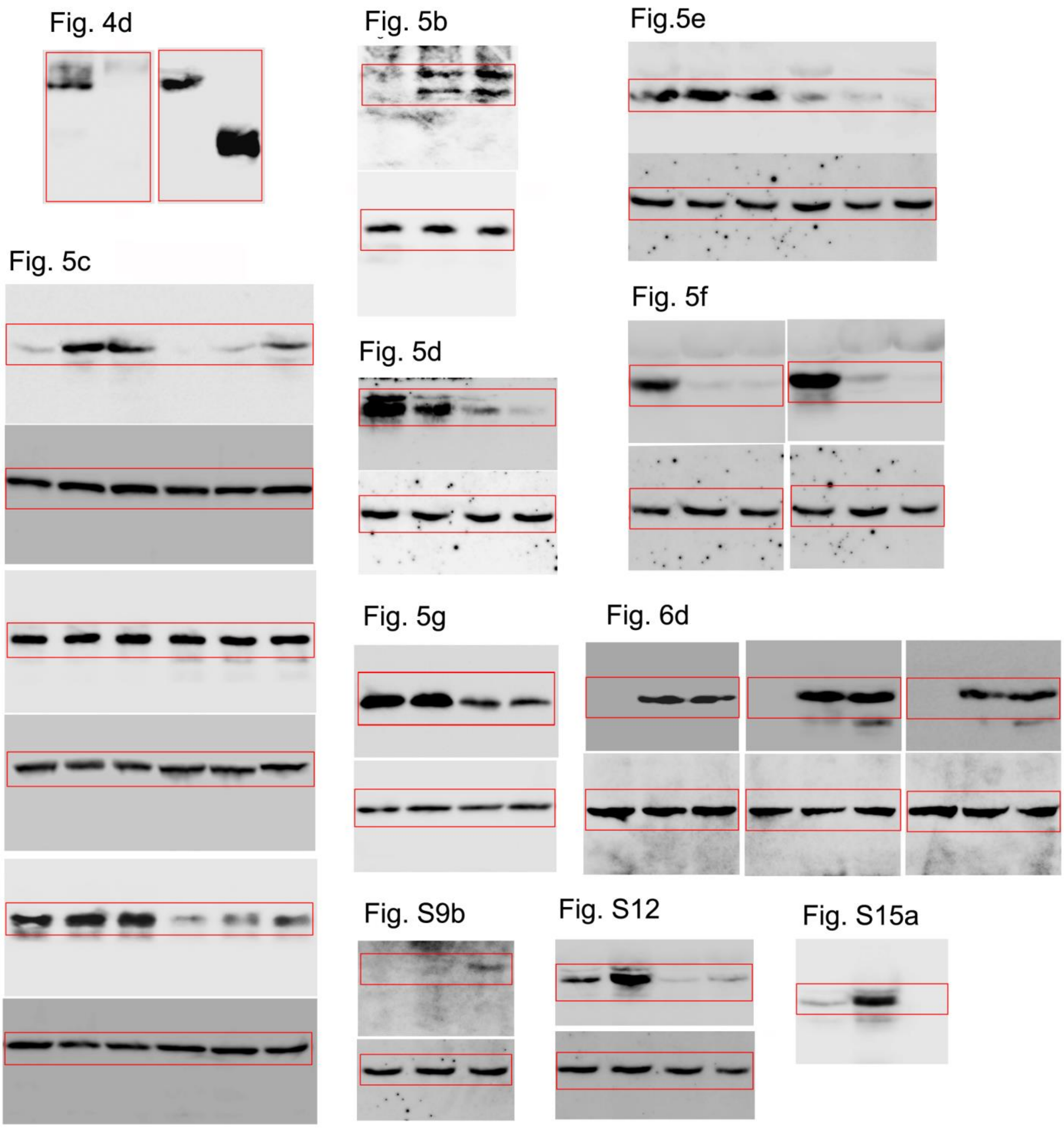

Supplementary Figure 21. Full view data of immunoblots partially shown in Fig. 4d, Fig. 5b~5f, Supplementary Fig. 9b, Supplementary Fig. 12 and Supplementary Fig.15a, respectively.

Supplementary Tables

Supplementary Table 1. GLS contents (μmol/g DW) in total aerial tissue of 16-day (16D) and 24-day (24D) old plants.

		16D	24D
Glucosinolate	3MSOP	0.16±0.04	1.52±0.07
	4MSOB	11.29±2.51	14.31±2.83
	5MSOP	0.46±0.10	0.69±0.14
	4MTB	1.75±0.39	2.02±0.41
	8MSOO	1.14±0.25	1.79±0.35
	I3M	2.46±0.55	3.38±0.0.67
	1MO-I3M	0.03±0.01	0.17±0.03
	**Total**	**17.29±3.85**	**23.87±4.48**

The aerial parts of wild-type (Col-0) at 16- and 24-day old plants in LD were used for LC-MS analysis of glucosinolates (GLSs).

Supplementary Table 2. GLS contents (μmol/g DW) in wild-type (WT) and *myb28 myb29* leaves at different growth stages.

		WT			*myb28 myb29*		
**Stage**		**16 D**	**20 D**	**24 D**	**16 D**	**20 D**	**24 D**
**Glucosinolate**	3MSOP	0.32±0.06	2.16±0.43	2.63±0.53	n.d.	n.d.	n.d.
	4MSOB	11.34± 2.27	18.04±3.61	21.21±4.25	n.d.	n.d.	n.d.
	5MSOP	0.70± 0.14	0.68±0.14	0.77±0.15	n.d.	n.d.	n.d.
	4MTB	2.13±0.43	2.75±0.55	3.08±0.62	n.d.	n.d.	n.d.
	8MSOO	1.28±0.26	1.81±0.36	2.10±0.42	n.d.	n.d.	n.d.
	I3M	3.11±0.62	3.22±0.64	3.51±0.70	1.79±0.36	2.11± 0.42	3.54±0.71
	1MO-I3M	0.07±0.01	0.11±0.02	0.35±0.07	0.04±0.01	0.19±0.04	0.38±0.08
	Total	18.95±3.79	28.77±5.76	33.65±6.73	1.83±0.37	2.30±0.46	3.92±0.79

Rapidly expanding leaves from the 16-, 20- and 24-day-old plants in LD were used for LC-MS analysis of glucosinolates (GLSs).

Supplementary Table 3. GLS contents (μmol/g DW) in wild-type and *cyp79b2 cyp79b3* leaves.

		WT	*cyp79b2 cyp79b3*
Glucosinolate	3MSOP	3.78±0.74	3.48±0.68
	4MSOB	19.82±3.87	20.64±4.03
	5MSOP	0.87±0.17	0.91±0.18
	4MTB	2.86±0.56	n.d.
	8MSOO	2.09±0.41	2.55±0.50
	**Total aliphatic**	**29.42±5.74**	**27.57±5.38**
	I3M	2.69±0.53	0.70±0.14
	1MO-I3M	0.59±0.12	n.d.
	**Total indole**	**3.29±0.64**	**0.70±0.14**

Rapidly expanding leaves from the 24-day-old plants in LD were used for LC-MS analysis.

Supplementary Table 4. GLS contents (μmol/g DW) in rapidly expanding leaves of 20- (20 D) or 40-day-old (40 D) plants of different genotypes in SD.

	Stage	20 D				40 D			
	genotype	WT	*35S:MIR156*	*SPL9:rSPL9*	*coi1-2*	WT	*35S:MIR156*	*SPL9:rSPL9*	*coi1-2*
Glucosinolate	3MSOP	2.88 ±0.58	2.96±0.59	2.27 ±0.45	0.98±0.20	5.16± 1.03	5.98±1.4	5.02±1.00	3.01±0.60
	4MSOB	13.69±2.74	13.82±2.76	10.65 ±2.13	4.04±0.81	28.89± 5.96	40.05±8.21	27.94±5.59	17.63±3.53
	5MSOP	0.98±0.20	1.03±0.21	0.49 ±0.10	0.21±0.04	1.10±0.22	1.28±0.26	1.03±0.21	0.78±0.16
	4MTB	7.55±1.51	4.91±0.98	5.14 ±1.03	3.38±0.68	8.07±1.65	10.10±2.02	7.29±1.46	9.12±1.82
	8MSOO	1.42±0.28	1.64 ±0.33	1.91 ±0.38	0.69±0.14	3.72±0.74	4.25±0.85	5.47±1.09	3.63±0.73
	I3M	4.62±0.92	10.20±2.04	3.08 ±0.62	1.30±0.26	5.42±0.88	11.45±1.49	5.77±1.15	1.82±0.36
	1MO-I3M	0.37±0.07	0.42±0.08	0.29 ±0.06	0.12±0.02	0.61±0.08	0.75±0.09	0.51±0.10	0.34±0.07
	Total	31.50±6.30	34.99±7.00	23.82±4.76	10.73±2.15	52.98±10.40	73.86±14.31	53.03±10.61	36.34±7.27

Plants were were grown in SD because the SPL9 over-expression plants (SPL9:rSPL9) were included in the analysis.

Supplementary Table 5. GLS contents (μmol/g DW) in the first two leaves at vigorous (Vigor) stage from the 14-day-old plants, or at senescent (Senescence) stage from the 28-day-old plants.

Stage		Vigor	Senescence
Glucosinolate	3MSOP	0.32±0.06	0.17±0.03
	4MSOB	11.64±2.33	2.28±0.46
	5MSOP	0.70±0.14	0.18±0.04
	4MTB	2.13±0.43	0.13±0.03
	8MSOO	1.28±0.26	0.47±0.09
	I3M	3.21±0.64	2.79±0.56
	1MO-I3M	0.07±0.01	0.05±0.01
	Total	19.35±3.87	6.08±1.22

Plants were grown in LD.

Supplementary Table 6. Possible mobilization of GLSs from the early senescence leaves to the newly initiated leaves.

		Intact	Wounded	Cut
Glucosinolate	3MSOP	2.93±0.51	4.67±0.82	1.25±0.22
	4MSOB	20.76±3.65	29.92±5.25	11.38±2.00
	5MSOP	0.60±0.10	0.93±0.16	0.57±0.10
	4MTB	3.03±0.53	2.62±0.46	1.32±0.23
	8MSOO	6.27±1.10	7.80±1.37	9.15±1.61
	I3M	2.52±0.44	4.47±0.78	3.88±0.68
	1MO-I3M	0.37±0.07	0.49±0.09	0.61±0.11
	Total	36.48±6.41	50.91±8.94	28.16±4.94

The 1st-5th leaves from the 20-day-old plants in LD were removed or wounded, four days later the four leaves initiated after starting of the treatments from the cut (Cut), wounded (Wounded) and intact (Intact) plants were harvested for detection of GLSs by LC-MS.

Supplementary Table 7. Primers used in this investigation.

Gene	Primer Sequence (5’-3’)	Purpose
LOX2	TTGGTGTGGTAACTACGATTGC<br>CACCAGCTCCAGCTCTATTCTT	qRT-PCR
VSP2	ACCCTCCTCTCTAGTATTCCC<br>ACTTGTACACCACTTGCCTCA	qRT-PCR
JAZ1	TTGGAGAAGAGAAAGGACAGAG<br>ATAGCAAGGGGATTTAGACAGG	qRT-PCR
TAT1	CCCTCAAAGACGTCAATGGT<br>ACACGACACGACAAGTCCAA	qRT-PCR
JAZ2	CAAAGGCACCATACCAAATAGA<br>GGGGGAGTTAAACAAACAAAAC	qRT-PCR
JAZ3	GCTGGAAGTAGCACAAACGGAC<br>AGGTTGCAGAGCTGAGAGAAGA	qRT-PCR
JAZ5	ATAATCACCTAACAAAGGAACA<br>GATAAGTCAGAAAAATCGAAAC	qRT-PCR
JAZ6	GAAGATCAAGAAACTGGGCAGC<br>TTGGTCAAAAGTAATGGAAGAA	qRT-PCR
JAZ10	TAATGAAGCAGCATCTAAGAAA<br>ATAAGCCAAATCCAAAAACGAA	qRT-PCR
JAZ11	GCTACGACTTCGGAAGCAGACA<br>AATCACAAACATGGAAAACAAT	qRT-PCR
JAZ12	CAACAAAAACCCTTACCCTACT<br>GCAAACATATCTGACCACTCCC	qRT-PCR
JAZ3HA	GCTGGAAGTAGCACAAACGGAC<br>CCCGAGCTCTTAAAATTCGAGCTCGGTG	qRT-PCR
CYP79B2	TAACGGTCACGAGAAGATTATG<br>GAGTTCTCTTTCCTTCTCTCCA	qRT-PCR
CYP79F1	AAAAGAGGTTACTCTGGTGGAA<br>CCAGTTAAACCCTTGAAGAAAC	qRT-PCR
CYP79F2	GATGATGGGAGACTAGGAAAAG<br>TTTCGACCCTCTCGTCTATTAT	qRT-PCR
CYP83A1	CAAGTGTTGAAGAAAGCTCAAG<br>TAAGGCTCTGAAGTAAGGAAGG	qRT-PCR
CYP83B1	GATGCAGATCTACAAAGACCAA<br>TGCCTTTAGGTAAGGGAGATTA	qRT-PCR
JAZ3	GCTCATATGGAGAGAGATTTTCTCG<br>CCCGAGCTCGGTTGCAGAGCTGAGA	Y2H
JAZ3δN	GCTCATATGGGTTCCTCTATGCCTCAAG<br>CCCGAGCTCGGTTGCAGAGCTGAGA	Y2H
JAZ3δC	GCTCATATGGAGAGAGATTTTCTCG<br>CCCGAGCTCATTGGTAGAACAAGAAACTG	Y2H

COI1	GCTCATATGGAGGATCCTGATATCAAG<br>CCCGAGCTCTGGCTCCTTCAGGACTC	Y2H /Y3H
JAZ1	CATGCCATGTCGAGTTCTATGGAATG<br>CCCCCCGGGTATTTCAGCTGCTAAACCGAGC	Y2H
JAZ2	CATGCCATGGAGATGTCGAGTTTTTCTGCCG<br>CCCCCCGGGTTACCGTGAACTGAGCCAAGC	Y2H
JAZ4	CATGCCATGGAGATGGAGAGAGATTTTCTCGG<br>CCCCCCGGGTTAGTGCAGATGATGAGCTGG	Y2H
JAZ6	CCCGAATTCATGTCAACGGGACAAGCG<br>CCCCCCGGGCTAAAGCTTGAGTTCAAGG	Y2H
JAZ7	CATGCCATGGAGATGATCATCATCATCAAAAAC<br>CCCCCCGGGCTATCGGTAACGGTGGTAAGG	Y2H
JAZ9	CATGCCATGGAGATGGAAAGAGATTTTCTGG<br>CCCCCCGGGTTATGTAGGAGAAGTAGAAG	Y2H
JAZ10	CATGCCATGGAGATGTCGAAAGCTACCATAG<br>CCCCCCGGGTTAGGCCGATGTCGGATAG	Y2H
JAZ11	CATGCCATGGAGATGGCTGAGGTAAACGGAG<br>CCCCCCGGGTCATGTCACAATGGGGC	Y2H
JAZ12	CATGCCATGGAGATGACTAAGGTGAAAGATGA<br>CCCCCCGGGCTAAGCAGTTGGAAATTCC	Y2H
JAZ3	CCGGAATTCATGGAGAGAGATTTTCTCG<br>CCCGTCGACGGTTGCAGAGCTGAGAGA	Y3H
SPL9	GTGGCGGCCGCAATGGAGATGGGTTCCAACTC<br>TCAGAGAGACCAGTTGGTATG	Y3H
SPL9	CCGGAATTCATGGAGATGGGTTCCAACTC<br>ACGCGTCGACTCAGAGAGACCAGTTGGTATG	Pull-down
JAZ3	ATGGAGAGAGATTTTCTCG<br>GGTTGCAGAGCTGAGAGA	Transgene
JAZ3δN	ATGGGTTCCTCTATGCCTCAAG<br>GGTTGCAGAGCTGAGAGA	Transgene
JAZ3δC	ATGGAGAGAGATTTTCTCG<br>ATTGGTAGAACAAGAAACTG	Transgene
JAZ3 promoter	CCGGAATTCTCATAGCGTGAGAGATGCGTTTA<br>CCCGAGCTCCTATAATAAAGACACAGCCCGCT	Transgene

1
